# Power-Based Multicomponent Training, Pelvic Floor Muscle Activation Instructions, and Psychoeducation for Urinary Incontinence, Balance and Fear of Falling in Older Women (FIRE-WOMEN): Study Protocol for a Randomized Controlled Trial

**DOI:** 10.3390/healthcare14162467

**Published:** 2026-08-10

**Authors:** Esther Ramos-Castellano, Teresa Mayordomo-Rodriguez, Ivan Chulvi-Medrano, Carlos Guillamo-Minguez, Pedro Gargallo-Bayo

**Affiliations:** 1 Doctoral School, Catholic University of Valencia San Vicente Martir, 46900 Valencia, Spain; 2 Department of Personality Psychology Treatment and Methodology, Faculty of Psychology, Catholic University of Valencia San Vicente Martir, 46900 Valencia, Spain; teresa.mayordomo@ucv.es; 3 Research Group in Prevention and Health in the Exercise and Sport (PHES), Department of Physical Education and Sport, University of Valencia, 46010 Valencia, Spain; ivan.chulvi@uv.es; 4 Department of Occupational Sciences, Faculty of Psychology, Catholic University of Valencia San Vicente Martir, 46900 Valencia, Spain; carlos.guillamo@ucv.es; 5 Exercise Intervention for Health Research Group (EXINH-RG), Department of Physiotherapy, University of Valencia, 46010 Valencia, Spain; pedro.gargallo@uv.es

**Keywords:** urinary incontinence, fear of falling, multicomponent training, psychoeducation, pelvic floor muscle, power training, older women

## Abstract

**Highlights:**

**What are the main findings?**

- This protocol describes a four-arm additive randomized controlled trial evaluating a multidisciplinary intervention combining power-based multicomponent training, pelvic floor muscle activation instructions, and psychoeducation in older women with urinary incontinence.
- The study will assess the additive effects of progressively combining pelvic floor muscle activation instructions and psychoeducation with power-based multicomponent training.

**What are the implications of the main findings?**

- Findings may support the implementation of multidimensional interventions targeting both urinary incontinence, balance and fear of falling in community-dwelling older women.
- The trial may contribute to the development of more comprehensive and integrated geriatric rehabilitation strategies.

**Abstract:**

**Background/Objectives**: Urinary incontinence (UI) and impaired balance are common geriatric syndromes that negatively affect health and quality of life in older women. However, the combined effects of power-based multicomponent training (PMT), pelvic floor muscle (PFM) activation instructions, and psychoeducational (PSY) support remain unclear. This study aims to evaluate the effects of a PMT, alone or progressively combined with PFM activation instructions and PSY support, on PFM function, UI symptoms, balance, fear of falling, and physical function in community-dwelling older women. **Methods**: This study describes the protocol for an 18-week, four-arm additive, parallel-group, assessor- and analyst-blinded randomized controlled trial with a 6-month follow-up. A total of 120 women aged 60–80 years with UI will be randomly assigned to four groups: (1) PMT only (EX), (2) PMT plus verbal PFM activation instructions (EXVERB), (3) PMT combined with PFM activation instructions and psychoeducational sessions (EXVERBPSY), or (4) a control group receiving educational sessions on geriatric syndrome prevention. Sessions will be conducted twice weekly for 18 weeks. The single primary endpoint is maximal PFM strength assessed by the manometric perineometry. Pre-specified secondary outcomes include additional PFM function measures, upper- and lower-limb (LL) strength and power, balance performance, fear of falling, quality of life, psychological well-being, and anthropometric variables. Assessments will be conducted at baseline, post-intervention, and 6-month follow-up. The trial is registered at ClinicalTrials.gov (NCT06839040). **Expected results**: This trial is expected to provide evidence on the effectiveness of a multidisciplinary intervention combining exercise, PFM activation strategies, and PSY support for reducing UI severity and improving balance performance and fear of falling in older women. **Conclusions**: This protocol describes a four-arm additive randomized controlled trial evaluating a multidisciplinary intervention targeting two highly prevalent and interrelated geriatric syndromes. The study may contribute to the development of more comprehensive exercise-based and PSY interventions for older women and may inform healthcare strategies aimed at promoting healthy ageing and improving the management of UI and fear of falling.

## 1. Introduction

The global aging population has led to a substantial increase in the prevalence of geriatric syndromes, particularly among women [1]. Age-related anatomical and functional changes contribute to impairments in postural control and neuromuscular function, increasing the risk of falls and urinary incontinence (UI) [2]. Fear of falling is highly prevalent among community-dwelling older adults and is strongly associated with the history of previous falls. Falls are a major public health concern and remain one of the leading causes of injury-related morbidity and mortality worldwide, prompting the development of international fall-prevention strategies [3,4].

UI is especially common in older women and is associated with significant psychological, social, and economic consequences [5]. Conservative interventions, particularly pelvic floor muscle (PFM) training, are recommended as first-line treatment and supported by high-quality evidence. Beyond its direct impact, growing evidence highlights a close relationship between UI and fall risk. Women with UI present a markedly higher likelihood of falling, which has been attributed to both intrinsic factors, such as reduced muscle strength and impaired postural control, and extrinsic factors related to functional limitations [6,7].

Biomechanical and neuromuscular studies have demonstrated an interaction between the PFM and key trunk and lower-limb (LL) muscles, including the transversus abdominis, gluteus maximus, and paraspinal musculature, underscoring the role of lumbopelvic stability in balance control [8]. Greater severity of UI has been associated with poorer balance performance and reduced postural stability in older women, particularly in those with diminished PFM strength. Consequently, international clinical guidelines increasingly recognize UI as a relevant component of multifactorial fall-risk assessment, reinforcing the need to address both conditions concurrently [9,10].

Although isolated PFM training remains the gold-standard intervention for UI, there is growing interest in complementary or more accessible strategies that may enhance functional outcomes and long-term adherence in older adults. General physical exercise has demonstrated beneficial effects on physical function and has been shown to contribute to the prevention and management of both UI and fear of falling [11,12,13,14]. In this context, multicomponent training programs (MTP), which combine strength, balance, mobility, and functional exercises within integrated sessions, are widely recommended to counteract age-related functional decline and reduce fall risk [15,16].

Within this framework, power-based MTP (PMT) represents a specific approach that emphasizes high-velocity force production to enhance functional performance [17]. In older adults, muscle power declines earlier and more rapidly than maximal strength and plays a critical role in tasks such as balance recovery and mobility. Power-oriented exercises have therefore been identified as particularly effective for improving functional capacity and fall-related outcomes in aging populations [18,19].

In addition to physical interventions, educational and PSY approaches may further enhance treatment effectiveness by addressing psychological barriers such as fear of falling, embarrassment, and low self-efficacy. These factors negatively influence physical activity participation and adherence, especially among older women with UI. Integrating PSY support within exercise-based interventions may promote behavioral change, improve adherence, and optimize functional outcomes [11,20].

The PSY component targets three interrelated psychological mechanisms expected to enhance intervention effects on the clinical outcomes. First, emotion regulation and acceptance-based strategies [21,22] address the shame, social anxiety, and experiential avoidance associated with UI, and the hypervigilance and activity curtailment driven by fear of falling, thereby facilitating sustained participation in the exercise programme [5,20,23]. Second, self-compassion training [24] reduces the negative self-evaluative responses triggered by UI episode, including self-criticism and feelings of loss of control—which amplify UI impact beyond its objective severity and are associated with poorer quality of life and reduced treatment engagement. Third, enhancement of self-efficacy for exercise adherence and UI self-management addresses a well-established mediator of treatment outcomes in older adults [25], given that UI-specific self-efficacy has been associated with greater adherence to PFM training and superior functional outcomes in comparable populations.

Although MTP and educational interventions have shown positive effects independently, existing studies have largely examined physical and psychosocial strategies in isolation [11,20,26]. This fragmented approach highlights the need for integrative, multidisciplinary interventions capable of simultaneously addressing UI, fall risk, and related psychological factors. Previous studies combining resistance exercise with PFM training have shown promising improvements in UI outcomes, supporting the integration of whole-body exercise and targeted PFM interventions [7]. Combining PMT with targeted PFM activation may optimize both LL function and PFM function, while PSY support may further enhance adherence, self-efficacy, and long-term behavioral change.

Beyond its established role in fall prevention, PMT may contribute to UI symptom reduction through four complementary mechanisms: (1) reflexive co-activation of PFM during closed kinetic chain resistance exercise [27,28]; (2) improvement of functional continence through gains in lower-limb power, walking speed, and sit-to-stand performance, which reduce UI episodes attributable to functional mobility limitations [7,29]; (3) improvement of body composition and reduction in intraabdominal pressure, a recognised risk factor for stress and mixed UI [17,30]; and (4) enhancement of lumbopelvic neuromuscular coordination between the transversus abdominis and pelvic floor, which constitutes a recognised mechanism of stress UI during physical effort [31,32]. These mechanisms provide an evidence-based rationale for combining PMT with targeted PFM activation instructions and psychoeducational support in a multidisciplinary intervention for UI in older women. Together, these complementary components are expected to provide greater overall benefits than each intervention delivered in isolation. To date, evidence evaluating combined models that integrate PMT, targeted PFM activation, and PSY support remains limited [7,15,16,20].

Beyond their clinical consequences, UI and fear of falling generate a substantial burden on healthcare systems due to their high prevalence, associated healthcare resource utilization, functional decline, and economic costs [3,4,5]. Therefore, evaluating integrated intervention models in community-dwelling older women is aligned with current healthcare priorities aimed at promoting prevention, patient-centred care, healthy ageing, and sustainable healthcare delivery. Given the substantially higher prevalence of UI among older women and the important contribution of female-specific anatomical and physiological factors, such as pregnancy, childbirth, and menopause, the present trial focuses exclusively on women. Restricting the study population to older women also provides a more homogeneous sample, reducing clinical heterogeneity associated with sex-specific mechanisms of UI and facilitating interpretation of the intervention effects. This study protocol aims to evaluate the effects of three combined intervention strategies over an 18-week period on PFM strength, LL function, balance, and UI symptoms in community-dwelling older women, as well as the maintenance of these effects at 6-month follow-up.

Based on the four-arm additive randomized controlled trial design and the multidimensional scope of the intervention, the following hypotheses are proposed:

**H1.**  *Older women receiving the most comprehensive intervention (EXVERBPSY) consisting of PMT combined with PFM activation instructions and PSY, will demonstrate greater improvements in PFM function, LL strength/power, balance and UI symptoms compared with the control group and groups receiving partial interventions.*

**H2.**  *All intervention groups will demonstrate progressively greater improvements than the control group, consistent with the additive contribution of each intervention component, with the greatest benefits expected in the EXVERBPSY group.*

**H3.**  *Improvements observed immediately post-intervention will be partially maintained at 6-month follow-up, with greater long-term retention observed in the EXVERBPSY group.*

**H4.**  *Among participants in the intervention groups, greater residualised improvements in psychological well-being (SF-36 Mental Component Summary score) and fear-of-falling-related self-efficacy (FES-I Short) from T0 to T1 will be positively associated with higher intervention adherence and with greater residualised improvements in the primary endpoint (maximal voluntary contraction pressure assessed by manometric perineometry) and the prespecified secondary outcomes at T1 and T2.*

## 2. Materials and Methods

### 2.1. Study Design

This study is designed as a four-arm additive, parallel-group, assessor- and analyst-blinded randomized controlled trial to evaluate the incremental contribution of PFM activation instructions and PSY when added to a PMT. The intervention will be conducted over an 18-week period, followed by a 6-month follow-up. The primary aim is to examine the combined effects of three intervention strategies on PFM function, LL performance, and balance in community-dwelling older women with UI.

Participants will be randomly allocated to one of four parallel groups: (1) a power-oriented PMT (EX); (2) exercise combined with verbal instructions to facilitate PFM activation during exercise (EXVERB); (3) exercise combined with PFM activation instructions and PSY support (EXVERBPSY); or (4) control group receiving educational sessions.

The intervention will be delivered over 18 weeks, with supervised sessions conducted twice weekly (55–60 min per session). Outcome assessments will be collected at baseline, immediately post-intervention (week 18), and at the 6-month follow-up to evaluate the persistence of effects. Data collection is planned from January 2026 to January 2027.

This study protocol has been prepared and is reported in accordance with the SPIRIT 2025 Statement (Standard Protocol Items: Recommendations for Interventional Trials) [33]. The results of the completed trial will be reported in accordance with the CONSORT 2025 Statement (Consolidated Standards of Reporting Trials) [34]. Analyses will follow the intention-to-treat principle. Ethical approval has been granted by the Ethics Committee of the Catholic University of Valencia (ID: UCV/2022-2023/123). The study is registered at ClinicalTrials.gov (Identifier: NCT06839040) and will be conducted in accordance with the Declaration of Helsinki.

The schedule of enrolment, interventions, and assessments, following the SPIRIT 2025 Statement, is presented in Supplementary Material Table S1.

### 2.2. Study Population

The study sample will include community-dwelling older women aged 60 to 80 years with a clinical diagnosis of UI. The International Consultation on Incontinence Questionnaire—Short Form (ICIQ-SF) [35] will be used to quantify UI symptom severity and impact at baseline and follow-up assessments. UI subtype (stress, urgency, or mixed) will be classified by a specialized physiotherapist according to the diagnostic criteria proposed by the International Continence Society (ICS) [36], based on participant’s medical history, symptom profile, and standardized clinical assessment prior to randomization. Cognitive function will be routinely assessed using the Mini Mental State Examination (MMSE). The Global Deterioration Scale (GDS) will only be used when administration of the MMSE is not feasible or considered inappropriate. When both instruments are available, the MMSE result will determine eligibility. Participants will be recruited from the Nau Gran program at the University of Valencia (Spain) and public centers affiliated with the City Council of Manises (Spain).

#### Selection Criteria

Detailed inclusion and exclusion criteria are presented in Table 1.

### 2.3. Recruitment Process and Screening

Participants will be recruited through informational sessions, posters, and community outreach activities conducted in collaboration with the Nau Gran program at the University of Valencia and public centers affiliated with the City Council of Manises. Interested individuals will be invited to attend an initial screening visit, during which the study objectives, procedures, potential risks, and benefits will be explained in detail.

The screening process will include demographic data collection, assessment of UI symptoms severity using the ICIQ-SF, cognitive screening using the GDS or MMSE, as appropriate, and a brief health history interview to verify inclusion and exclusion criteria, including the presence of a clinical diagnosis of UI. Only participants meeting all inclusion criteria will be enrolled and randomized. Written informed consent will be obtained prior to enrollment, and participants may withdraw at any time.

### 2.4. Sample Size Calculation

Sample size was estimated a priori using G*Power 3.1 [37]. Maximal voluntary contraction pressure (mmHg), assessed by manometric perineometry, was selected as the single primary outcome because it provides an objective and quantitative measure of PFM strength and is the outcome most directly related to the primary study hypothesis.

Previous randomized controlled trials involving women with UI have shown that vaginal manometric measures of PFM strength and endurance are responsive to exercise-based interventions incorporating specific or voluntary PFM contractions. In particular, Gonzaga et al. [38] reported improvements in manometric measures of PFM strength and endurance following PFM training and Pilates exercise performed with voluntary PFM contraction in postmenopausal women with UI. However, because previous studies are characterized by relatively small sample sizes and heterogeneous interventions and do not provide either a directly applicable standardized effect size or an established clinically important difference for the planned group-by-time comparison, Cohen’s f = 0.24 was selected as a conservative a priori planning assumption rather than an empirically derived effect estimate [39,40].

The calculation was based on a repeated-measures ANOVA model (within-between interaction; four groups; three time points: T0, T1, T2; two-tailed α = 0.05; statistical power = 1 − β = 0.80; assumed correlation among repeated measures r = 0.50; and Cohen’s f = 0.24). Under these assumptions, the minimum analytical sample required was estimated at 96 participants. Nevertheless, the planned recruitment target was set at 120 participants (30 per group). This target exceeds the minimum sample size required by the formal power calculation and was selected to preserve balanced allocation across the four intervention groups, accommodate anticipated attrition and incomplete follow-up data, and improve the precision and stability of the estimated longitudinal treatment effects obtained from the planned linear mixed-effects models (LMM). The anticipated attrition rate was conservatively estimated at 20%, based on participant retention reported in comparable randomized exercise-based interventions involving older women with UI [7,41,42].

The primary analysis will be performed using LMM framework consistent with the longitudinal design described in the Statistical Analysis Section 2.8. Although the analytical model differs from the repeated-measures ANOVA framework in G*Power 3.1, the latter was used to provide a transparent and reproducible a priori approximation of the required sample size under a balanced design and the stated correlation assumptions. Any difference in statistical power between this approximation and the final LMM is acknowledged as a limitation of the sample size estimation [43].

### 2.5. Randomization and Blinding

Randomization will be performed after completion of baseline assessment and informed consent procedures. An independent researcher not involved in participant recruitment, intervention delivery, outcome assessment, or data analysis will generate the allocation sequence using the Sealed Envelope^TM^ (London, UK) online randomization tool, with a 1:1:1:1 allocation ratio.

Allocation concealment will be ensured using sealed, opaque, consecutively numbered envelopes prepared according to the generated sequence. The envelopes will be kept by an independent researcher and opened sequentially only after each participant has completed baseline assessments and provided written informed consent. Outcome assessors and data analysts will remain blinded to group allocation throughout the study.

Due to the nature of the intervention, participants and intervention providers cannot be blinded. Several measures will be implemented to minimize performance and detection bias. First, participants will be informed that they will be randomly assigned to one of several intervention approaches. However, they will not be provided with information regarding the specific study hypotheses or the intervention received by the other groups. Participants will be told that the study evaluates different approaches to improving physical function and urinary control in older women, without reference to the specific components assigned to each group. Second, exercise sessions for different intervention groups will be scheduled at separate time slots throughout the week, ensuring that participants from different groups do not share waiting areas, changing rooms, or common spaces during study activities. This separation of session schedules minimizes the risk of contamination and inadvertent unblinding. Third, participants randomized to the control group will receive their educational sessions in a separate setting and at different times from the exercise groups.

Given the multidimensional nature of the intervention, blinding of the intervention supervisor and the physiotherapist delivering the sessions is not possible. However, a standardized supervision checklist will be used to ensure intervention fidelity across sessions and minimize intervention-delivery drift.

**Figure 1:**
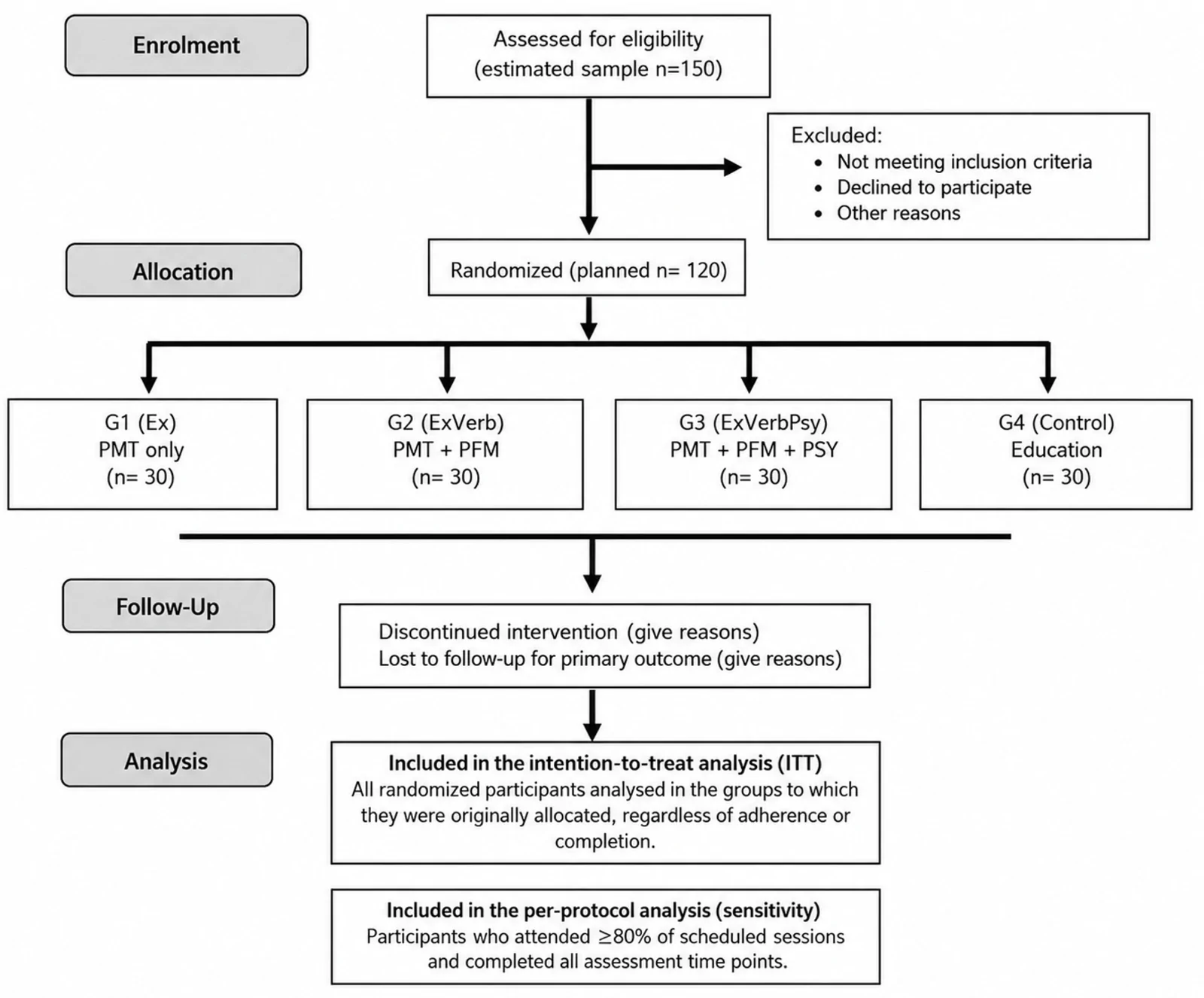
Anticipated participant flow throughout the study, including screening, randomization, allocation, follow-up, and analysis. Abbreviations: PMT: power-based multicomponent training; PFM: pelvic floor muscle; PSY: psychoeducational intervention; Ex: exercise group; ExVerb: exercise plus verbal pelvic floor muscle activation instructions; ExVerbPsy: exercise plus verbal pelvic floor muscle activation instructions and psychoeducation.

A flow diagram describing the anticipated participant progression throughout screening, randomization, allocation, follow-up, and analysis phases is presented in Figure 1.

### 2.6. Interventions

The intervention will consist of 36 supervised multicomponent exercise sessions, and, for one study arm, six structured PSY sessions delivered over 18 weeks. Exercise sessions will be conducted twice weekly on non-consecutive days (55–60 min/session) and will target balance, power, strength, aerobic capacity, agility/coordination, and flexibility with progressive intensity. Sessions will be led by a physiotherapist specialized in geriatrics (five years of experience), with methodological oversight by a research supervisor using a standardized supervision checklist to ensure fidelity and safety.

Participants will be allocated to one of the four groups:

- G1: (EX) PMT only.
- G2: (EXVERB) EX plus verbal cues to facilitate PFM contractions during balance and strength exercises.
- G3: (EXVERBPSY) EXVERB plus six structured PSY sessions delivered during the intervention period.
- G4: (Control Group) Participants will receive four educational sessions (one per month, 45–60 min per session) focused on the prevention of geriatric syndromes and instructions to maintain their usual physical activity. The sessions will address topics related to breathing control, postural control, balance and fall prevention, and pelvic floor (PF) health. After study completion, control participants will be offered the opportunity to receive the intervention components not undertaken during the initial study phase.

#### 2.6.1. Adherence and Intervention Fidelity

Adherence will be promoted through regular participant contact (telephone and/or in person), flexible scheduling, and reminder messages prior to assessments. Attendance of ≥80% of scheduled sessions will be considered adequate adherence. Attendance, progression variables (e.g., elastic-band resistance and grip width), and any adverse events (AE) will be recorded throughout the intervention. Reasons for discontinuation will be documented, and outcome data will be collected at each assessment time point whenever feasible, in accordance with the intention-to-treat principle.

#### 2.6.2. General Aspects of the Exercise Protocol

Training will be delivered in indoor multipurpose rooms at the Faculty of Physiotherapy (University of Valencia) and the Cultural Center of Manises (Valencia, Spain). Prior to the intervention, participants allocated to the exercise intervention groups (EX, EXVERB, and EXVERBPSY) will complete two familiarization sessions over a two-week period to ensure the correct execution of the MTP, including movement technique, movement velocity, breathing patterns, and intensity regulation. Participants allocated to the EXVERB and EXVERBPSY groups will additionally complete two familiarization sessions as part of the PFM activation intervention. The first session will provide standardized educational information on PF anatomy and function, while the second session will include individualized instruction on correct PFM activation using biofeedback to enhance motor learning and control. Consequently, participants in the EX group will complete two familiarization sessions, whereas participants in the EXVERB and EXVERBPSY groups will complete four familiarization sessions in total. Participants allocated to the control group will not receive familiarization sessions.

Each session will follow a standardized structure based on American College of Sports Medicine (ACSM) recommendations [44]: (1) warm-up (≈5 min; joint mobility and low-intensity aerobic activity); (2) main phase (≈50 min; balance, power, strength, aerobic, agility/coordination, and flexibility components); and (3) cool-down (≈5 min; breathing and stretching). The duration assigned to each training component is approximate and includes the time required for exercise transitions, standardized verbal instructions, PFM cueing (when applicable), and the prescribed recovery periods between sets and exercises, thereby ensuring that the total duration of each supervised session remains within the planned 55–60 min. Training will be progressed according to specificity and progressive overload principles, aligned with evidence-based recommendations for fall prevention [45] and current clinical practice guidelines for PFM training in women [11,12,46].

Exercise intensity will be adjusted weekly by modifying elastic band resistance (TheraBand^®^ CLX Loops), grip width, exercise duration, and postural demands according to the participant’s perceived exertion (OMNI-RES for elastic resistance) and their ability to complete the prescribed repetitions with correct technique [47]. The percentages presented in the intervention tables should be interpreted as approximate contextual descriptors of relative training intensity and do not correspond to directly measured one-repetition maximum (1RM) values for elastic resistance. Participants will perform exercises in a consistent order, alternating upper- and LL activities to manage fatigue [48]. Participants will be asked to maintain usual dietary and physical activity habits and to avoid other structured exercise programs during the study period.

Detailed progression of the multicomponent program across the 18-week intervention is provided in Table 2.

#### 2.6.3. Mobility/Warm-Up Component

Dynamic joint mobility exercises targeting the main joints involved in the training session (e.g., spine, shoulders, wrists, hips, knees, and ankles) will be performed, followed by low-intensity aerobic activity (40–55% of maximum heart rate), such as marching in place, light walking, or step-based movements. This warm-up aims to increase body temperature, enhance joint range of motion, and prepare the neuromuscular system for subsequent balance, strength, and power exercises. This warm-up protocol is consistent with current exercise prescription recommendations for older adults [44,49].

#### 2.6.4. Balance Training Component

**Figure 2:**
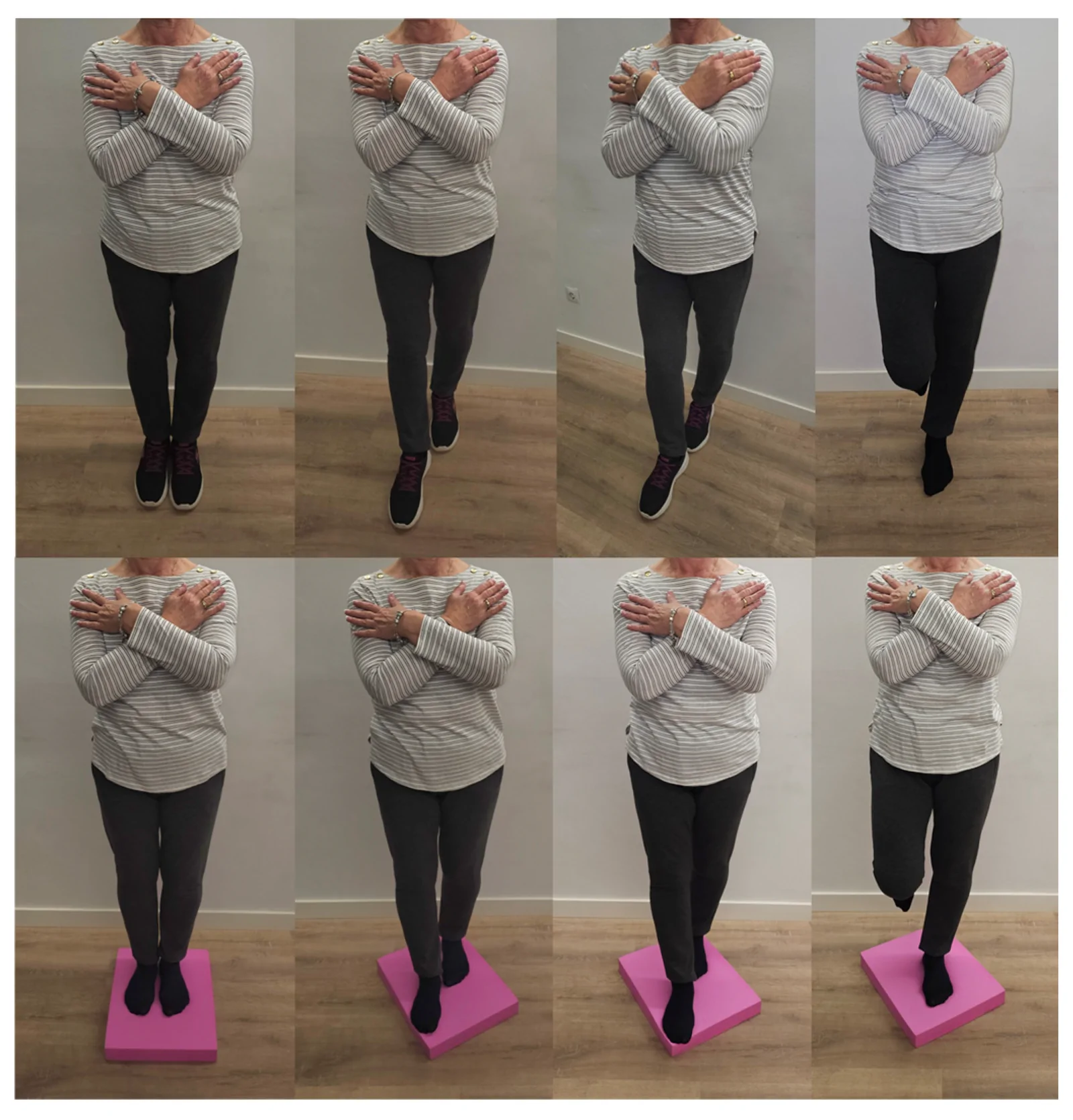
Balance exercises.

The balance component will primarily target standing balance, with progressive increases in task difficulty by manipulating base of support, support surface (stable/unstable), visual input, center-of-mass position, and time under tension. Standing balance exercises were specifically selected because the intervention aims to improve functional postural control during weight-bearing activities, which are most closely associated with mobility limitations and falls in community-dwelling older adults. Safety will be ensured by positioning a chair or stable support adjacent to each participant when required. The balance progression by phase is described in Figure 2 and summarized in Table 3.

#### 2.6.5. Power-Strength Training Component

Power training will include two LL exercises (seated knee extension and standing hip extension) performed using CLX looped elastic bands (TheraBand, The Hygenic Corporation, Akron, OH, USA). Participants will be instructed to execute the concentric phase with maximal intentional velocity, followed by a brief isometric pause (1 s) and a controlled eccentric phase (2 s) to ensure technical accuracy and safety.

Training intensity will be regulated by a combination of objective criteria (hand-grip position, band color; maximum stretch 300% of the resting band length) and subjective effort, monitored using the rating of perceived exertion recorded after the first repetition of each set (RPE-1). The target power training intensity will correspond to an RPE of 5–6 throughout the intervention, with band tension progressively adjusted to maintain the prescribed level of perceived exertion while enabling to perform the concentric phase with maximal intended velocity. Participants will be instructed to execute each repetition as fast as possible during the concentric phase while maintaining correct technique, as movement velocity has been identified as a key determinant of improvements in physical function [18,19].

Intensity will be monitored during each session using the OMNI-RES RPE scale for elastic band resistance exercise [47], which constitutes the primary real-time operational tool for intensity control. Exercise intensity will be adjusted by modifying elastic band resistance, grip width, exercise duration, and postural demands according to participants perceived exertion and their ability to complete the prescribed repetitions with correct technique. The %1RM values reported in the intervention tables should be interpreted solely as approximate contextual descriptors of relative training intensity to facilitate comparison with the broader resistance-training literature and do not correspond to directly measured one-repetition maximum (1RM) values for elastic resistance [47].

Training intensity will progress across the 18-week intervention following a structured periodization scheme (Table 4). During weeks 1–2, intensity will be set at approximately 60% of estimated 1RM (initial RPE: iRPE 2–3; final set RPE: fRPE 5–6). During weeks 3–7, intensity will increase to approximately 65% of estimated 1RM (iRPE 3–4; fRPE 5–6). From week 8 onwards (Weeks 8–18), intensity will be maintained at approximately 70% of estimated 1RM (iRPE 3–5; fRPE 5–6), consolidating neuromuscular adaptations prior to post-intervention assessment. These values are consistent with the progressive scheme shown in Table 4.

**Figure 3:**
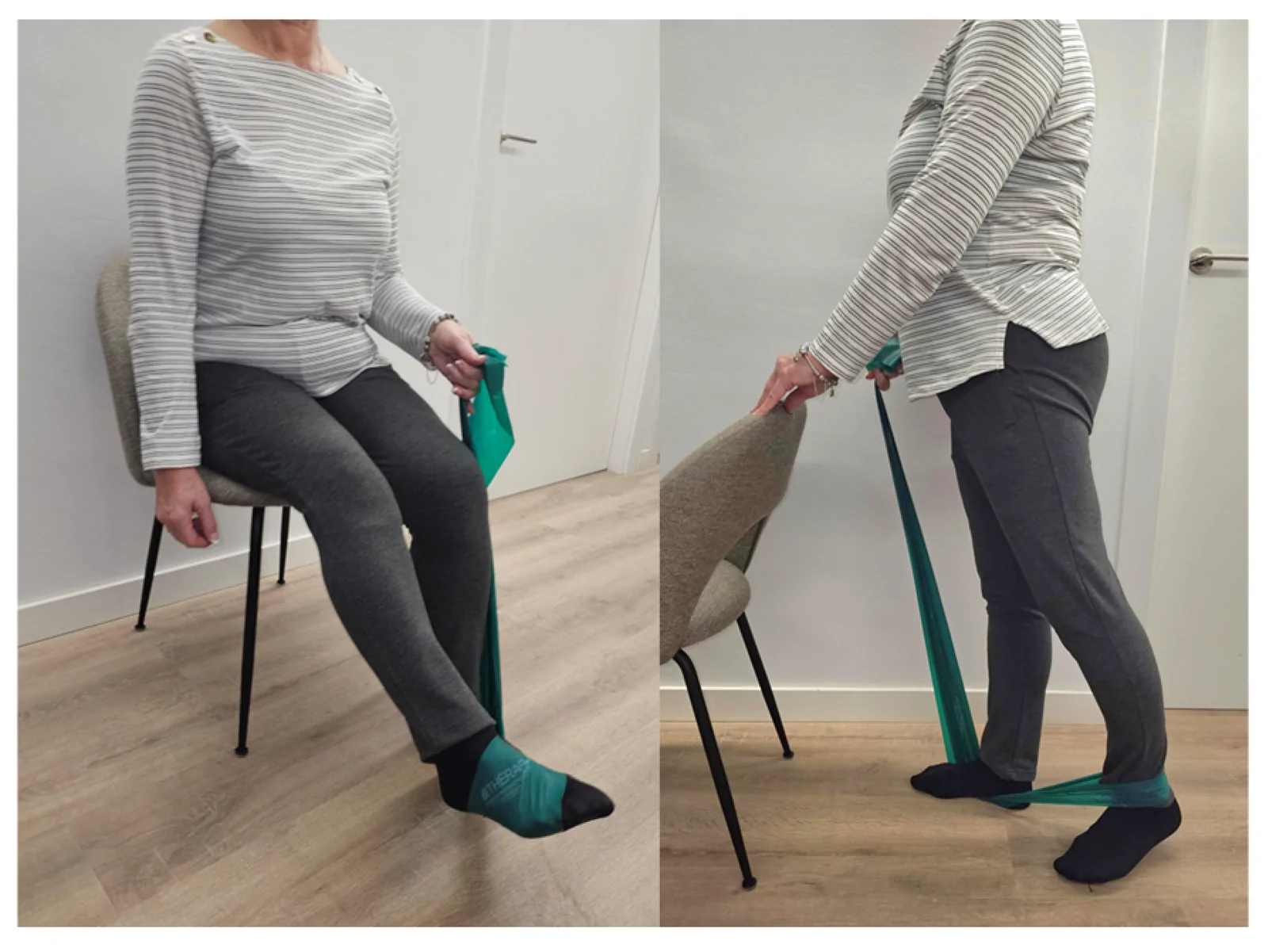
Power-strength exercises.

To consolidate technique, participants will perform 10 repetitions per set during the first two weeks and 8 repetitions thereafter. The number of sets per exercise will progress from 2 during the first 7 weeks (weeks 1–7) to 3 from week 8 until the end of the intervention (weeks 8–18), with 90 s of active recovery between sets (rhythmic swinging of the extremities, coordinative movements, along with cognitive tasks without the use of elastic bands) and 60 s of rest between exercises for hydration or sweat wiping. The power-strength exercises are described in Figure 3 and summarized in Table 4. The detailed exercise prescription and progression across the intervention period are presented in Table 5.

#### 2.6.6. Strength Training Component

This block will initially consist of four strength exercises performed in sequence during the first 7 weeks of the intervention: lateral trunk inclinations, squat with shoulder press, standing hip abduction and standing shoulder abduction in the scapula plane using CLX looped elastic bands (TheraBand, Hygenic Corporation, Akron, OH, USA). From week 8 onwards, lateral trunk inclinations will be removed, and the programme will consist of the three strength exercises: squat with shoulder press, standing hip abduction and standing shoulder abduction in the scapula plane. Training intensity will be adjusted by band tension (grip position, band color, number of bands, elongation restricted to a maximum of 300% of the resting length) and monitored with RPE at the end of the set. Participants will perform 10 repetitions per set during the first two weeks and 8 repetitions thereafter. The number of sets will progress from two during the first 7 weeks (weeks 1–7) to three from week 8 until the end of the intervention (weeks 8–18), at a target RPE of 5–9.

Training intensity in the strength block will be regulated using the RPE recorded at the end of each set, following conventional resistance training practice. This differs from the power training block, in which RPE will be assessed after the first repetition (RPE-1) to preserve movement velocity throughout the set. In the strength block, end-of-set RPE will reflect the accumulated neuromuscular fatigue generated across all repetitions, which is the relevant stimulus for strength adaptations. All exercises will be performed with a controlled concentric phase (1–2 s), a brief isometric pause (1 s), and a controlled eccentric phase (2–3 s), ensuring mechanical tension throughout the full range of motion. During the final phase of the programme (weeks 13–18), in which intensity will reach 80% of the estimated 1RM (RPE 8–9), the supervising physiotherapist will actively monitor technique execution on a repetition-by-repetition basis. Special attention will be paid to the squat with shoulder press, where accumulated fatigue may result in lumbar hyperextension during the overhead pressing phase or loss of knee alignment during the squatting phase, both of which represent potential injury risks in older adults. Sets will be terminated if technical failure is observed, regardless of whether the prescribed number of repetitions has been completed.

The number of sets per exercise will progress from two during weeks 1–7 to three during weeks 8–18, with 90 s of active recovery between sets (rhythmic swinging of the extremities, coordinative movements, along with cognitive tasks without the use of elastic bands) and 60 s of rest between exercises for hydration or sweat wiping. The introduction of low-intensity active pauses with engaging exercises will be used as a strategy to improve adherence to the program, in addition to increasing caloric expenditure and improving the cardiovascular system.

The detailed exercise prescription and progression across the intervention period are presented in Table 6.

**Figure 4:**
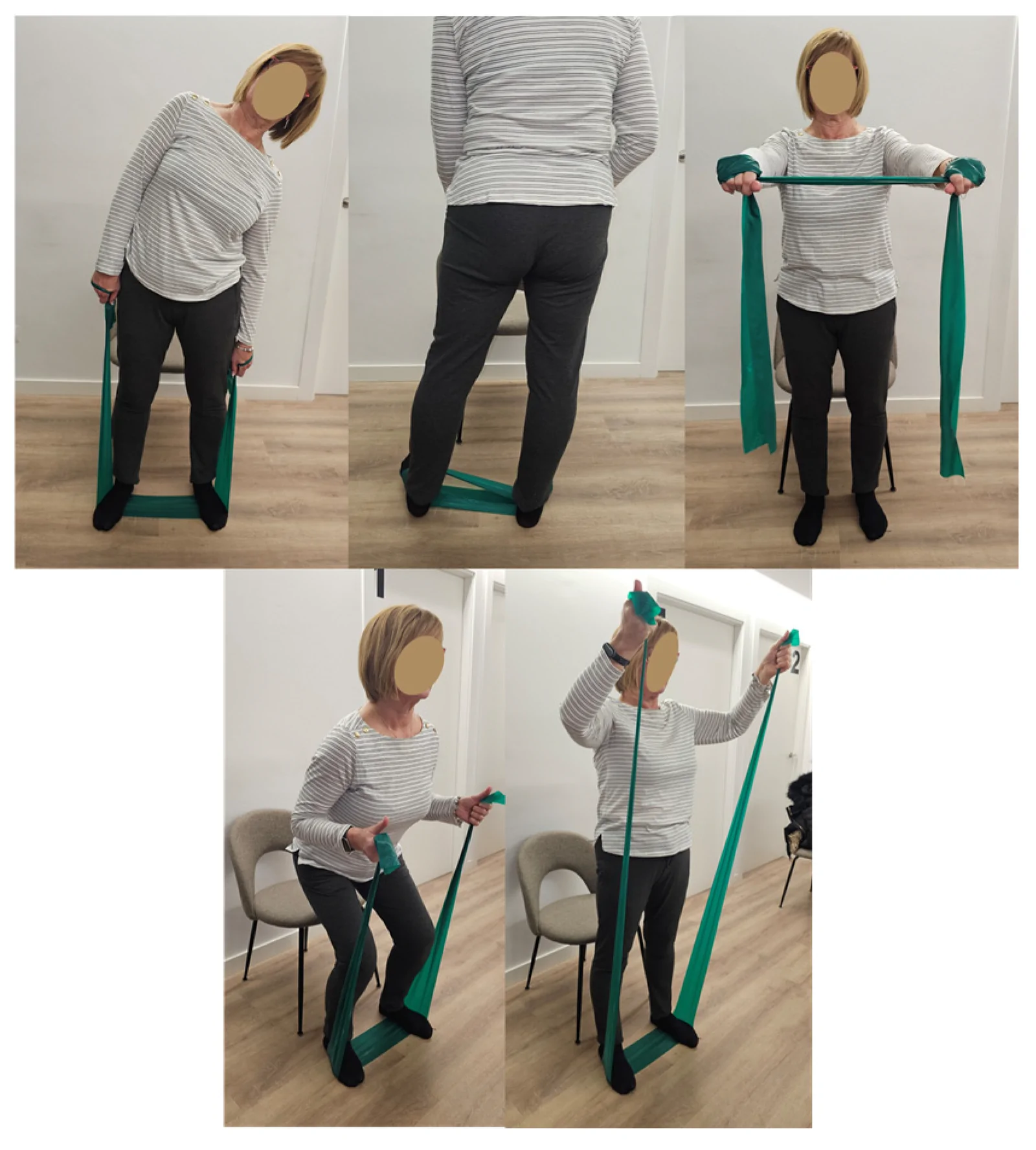
Strength exercises.

Exercise descriptions, key cues, and safety considerations are provided in Figure 4 and Table 7.

#### 2.6.7. Aerobic and Coordination Training Component

The aerobic component will combine stationary and locomotor tasks designed to reflect everyday mobility demands and reduce fall risk, incorporation multidirectional gait patterns, changes in tempo, and brief accelerations/decelerations. Aerobic intensity will progressively increase from approximately 65% to 85% of age-predicted maximum heart rate and will be monitored using the Borg RPE scale (6–20), targeting values between 11 and 15 [50,51].

**Figure 5:**
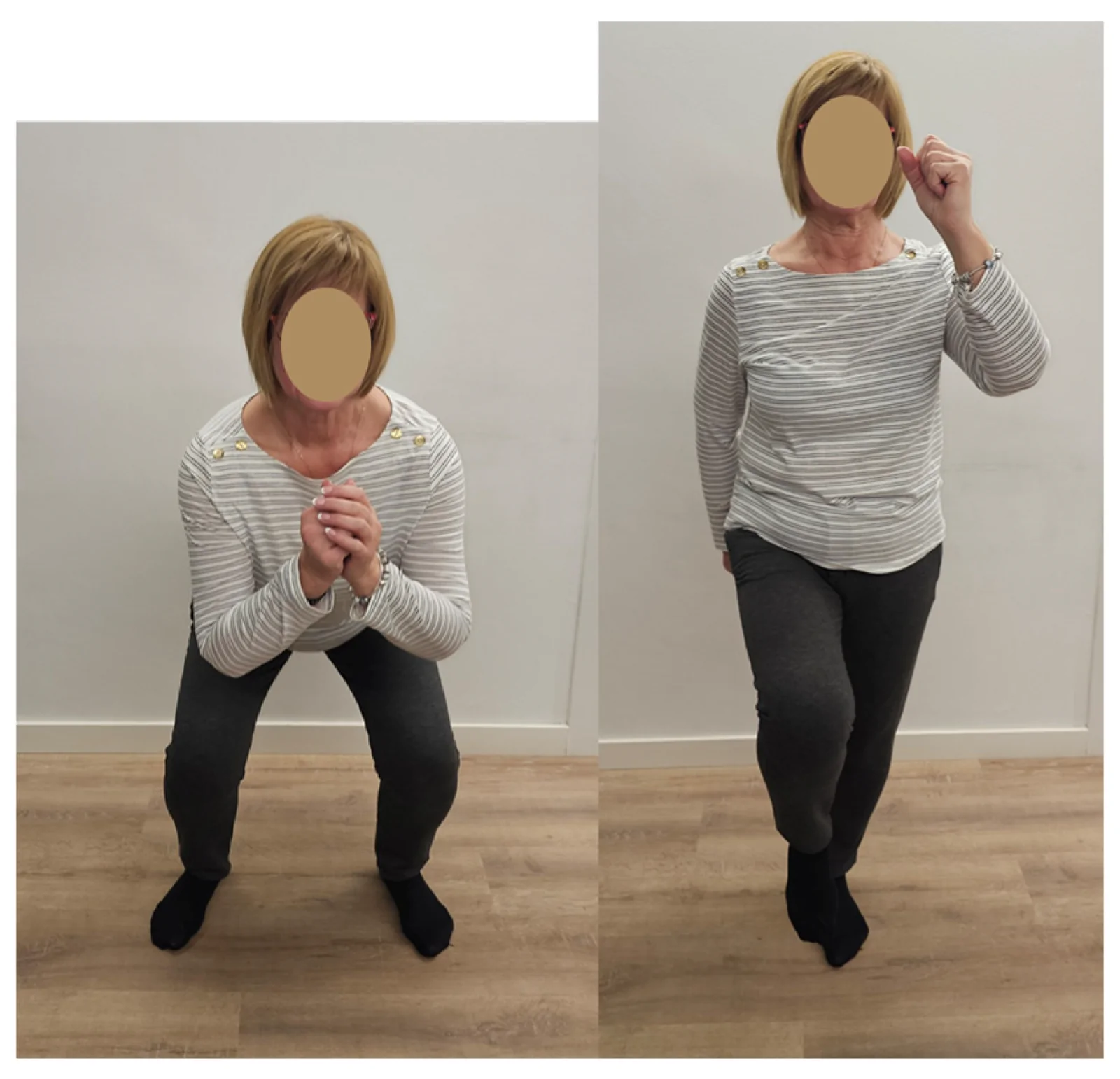
Aerobic and coordination exercises.

To differentiate the aerobic component from the warm-up phase, the aerobic block will incorporate more complex locomotor and coordinative task performed at moderate-to-vigorous intensity. Coordination and dual-tasks activities will be interleaved during recovery periods of the power, strength and aerobic blocks. These activities will include reaction-time drills, dance-based sequences, obstacle negotiation, and progressively complex cognitive flexibility tasks, as illustrated in Figure 5 and Table 8.

#### 2.6.8. Flexibility and Relaxation Training Component

Participants will perform diaphragmatic breathing exercises and static stretching targeting the main muscle groups. One repetition of each stretch will be held for approximately 20 s, and participants will be instructed to perceive a mild, non-painful stretching sensation during each exercise.

**Figure 6:**
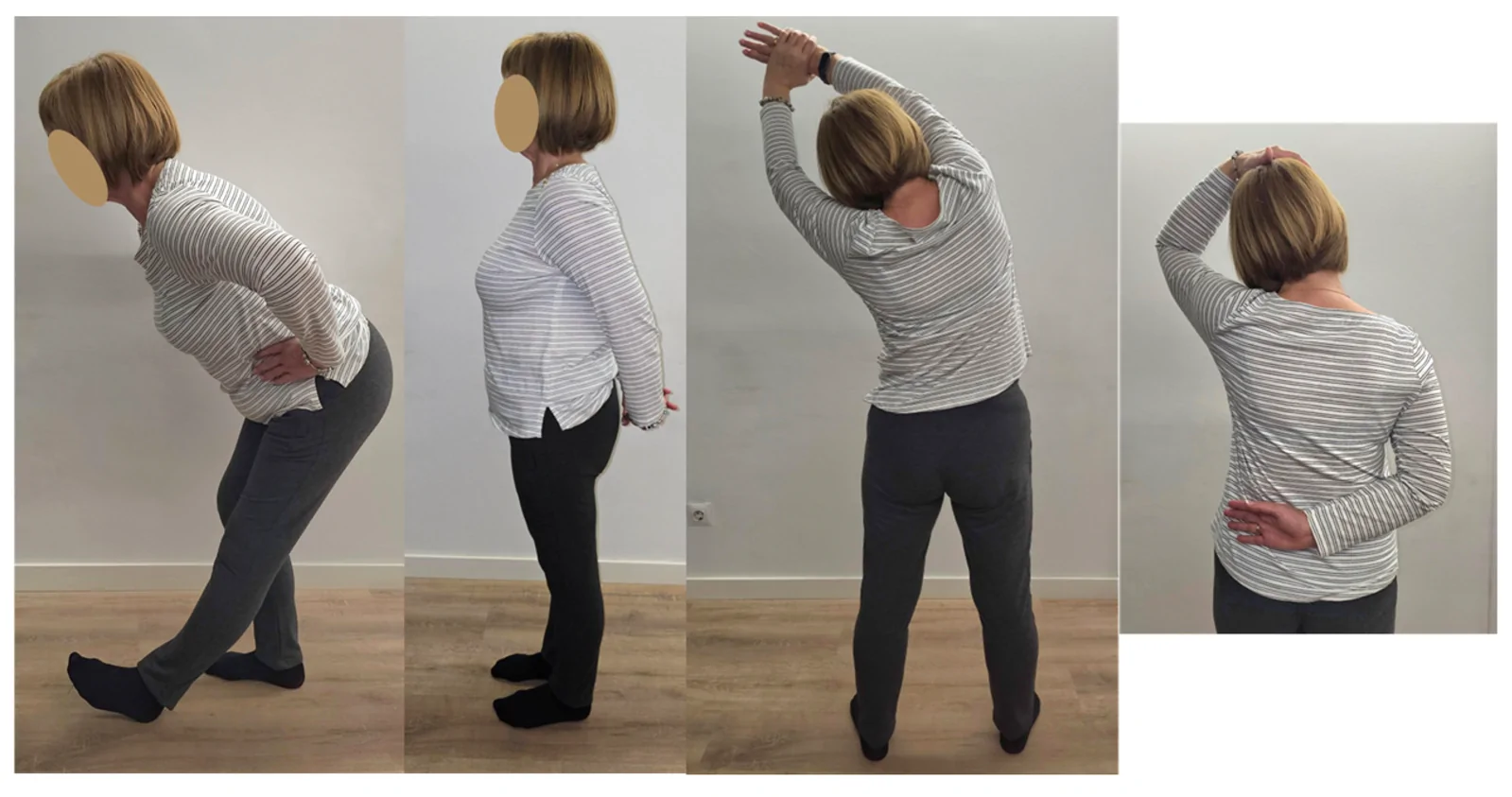
Flexibility exercises.

The flexibility exercises included in the program are illustrated in Figure 6.

#### 2.6.9. Pelvic Floor Intervention

Participants allocated to EXVERB and EXVERBPSY will standardize instruction to perform voluntary PFM contractions integrated into balance, strength, and power exercises to promote functional coordination of the PF during global motor tasks. The PFM component will include both slow (8–10 s) and fast (1–3 s) contractions. Initially, participants will practice correct PFM activation in stable positions to support selective activation and awareness. PFM activation will then be progressively integrated into standing balance tasks, emphasizing coordinated co-activation with diaphragmatic breathing and lumbopelvic control. During power and strength blocks, fast PFM contractions will be cued during functional exercises performed under increased mechanical and postural demands to reinforce coordinates PFM activation [42,52].

#### 2.6.10. Psychoeducation Intervention

The PSY sessions will be delivered by a clinical psychologist specialized in gerontology once or twice per month (six sessions total) during the 18-week intervention period. The sessions will address emotional awareness, cognitive flexibility, and understanding and regulation of emotions (e.g., stress, fear, anxiety), as well as pain and sexuality-related concerns. Session development will be informed by the Unified Protocol for Transdiagnostic Treatment of Emotional Disorders [21], supplemented with selected components from Acceptance and Commitment Therapy [22] and self-compassion-based approaches [24]. The primary aim is to improve emotion regulation through identification of emotional responses, selection of appropriate regulation strategies, and implementation of these strategies in daily life. The content and structure of the PSY sessions are summarized in Table 9. The psychoeducational sessions are theoretically grounded in three mechanisms linking psychological content to the primary clinical outcomes of this trial: (1) emotion regulation and acceptance techniques reduce experiential avoidance associated with UI and fear of falling, promoting valued-action engagement and sustained adherence to the exercise programme [21,22]; (2) self-compassion strategies reduce the psychological amplification of UI impact caused by self-critical responses to leakage episodes, improving quality of life and treatment engagement independently of symptom severity [24]; and (3) self-efficacy enhancement targets exercise adherence and UI self-management behaviours, which are established mediators of functional outcomes in older adults with UI [25]. Home-practice materials will be provided, and audio recordings of guided practices will be made available upon request to facilitate continued practice. Between-session tasks and reflective exercises will be prescribed and subsequently reviewed during the following sessions to monitor adherence and facilitate skill consolidation. Session materials and reminders will also be shared through a communication group to enhance intervention fidelity and support continued engagement.

**Figure 7:**
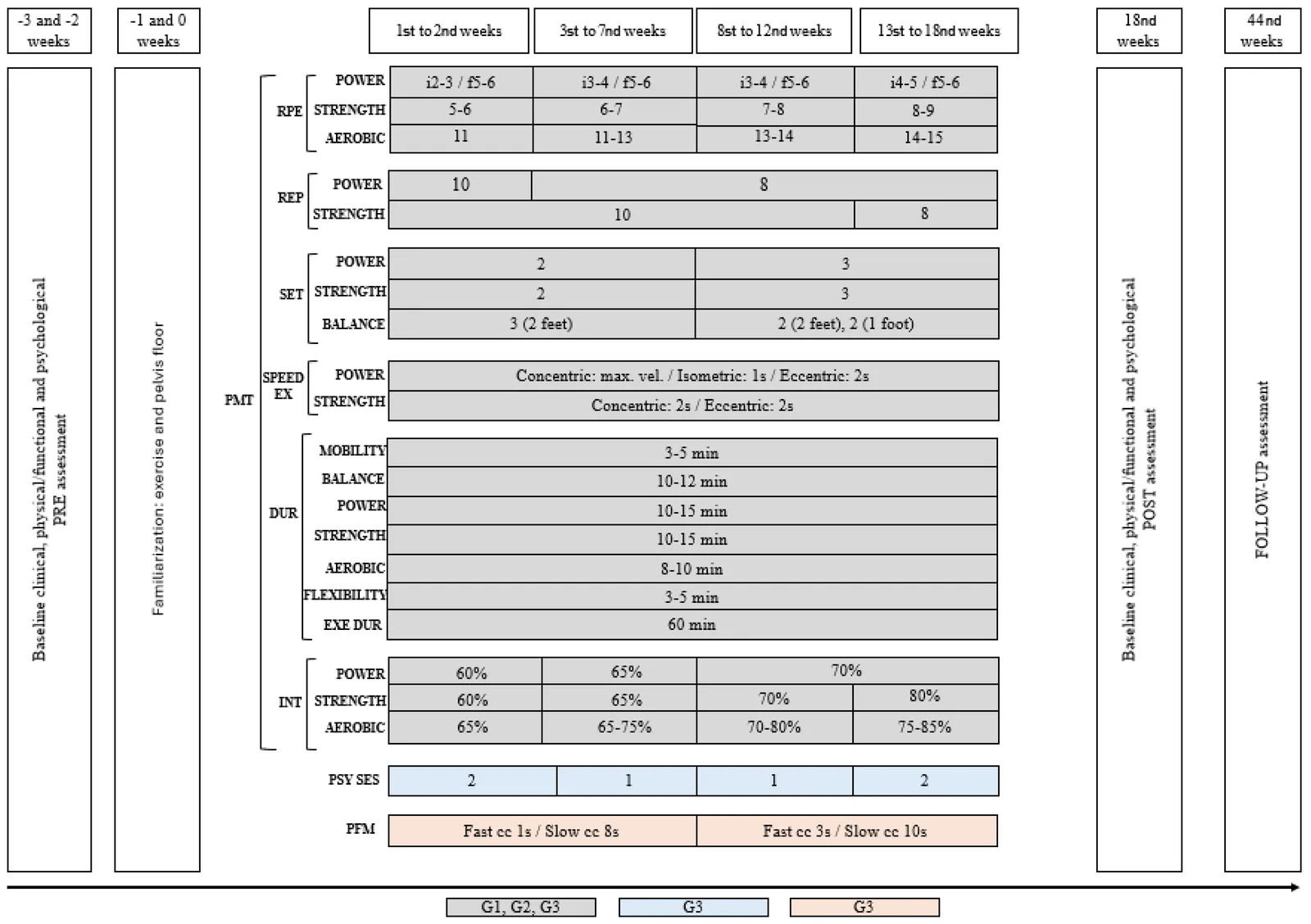
Experimental design. Abbreviations: PMT: power-based multicomponent training; PFM: pelvic floor muscle; PSY: psychoeducational intervention; EX: exercise; i: initial; f: final; s: seconds; min: minutes; CC: contractions; DUR: duration; INT: intensity; REP: repetitions; RPE: rating of perceived exertion; SES: sessions; SET: sets. Note: Component durations are approximate and may vary slightly across sessions. Recovery periods between sets and exercises are included within the planned total session duration of approximately 55–60 min.

An overview of the experimental design and intervention arms is provided in Figure 7.

To summarize, the experimental design with the training and assessment characteristics can be observed in Figure 7.

#### 2.6.11. Follow-Up and Co-Interventions

During the 6-month follow-up period, no supervised intervention sessions will be provided. Control participants will be offered access to the intervention components only after completion of the final follow-up assessment. Participants from all groups will be instructed to maintain their usual lifestyle habits, and self-reported physical activity levels will be monitored throughout the follow-up period. In addition, any co-interventions potentially affecting study outcomes, including physiotherapy treatments, PFM rehabilitation, or changes in UI related medication, will be recorded and considered during data interpretation.

### 2.7. Study Variables

Outcome measures will assess physical/functional and psychological domains. Sociodemographic and clinical variables (age, anthropometrics, obstetric history [number of pregnancies and vaginal deliveries], menopause onset, comorbidities, medication use, and lifestyle factors) will be collected at baseline. Current medications potentially affecting urinary function (e.g., diuretics and antihypertensive medications) will also be recorded. All outcome measures will be assessed at baseline, post-intervention (18 weeks), and at 6-month follow-up.

#### 2.7.1. Primary Outcome Measures

- Primary end-point: Pelvic floor muscle strength

Maximal PFM strength will be assessed using vaginal manometric perineometry, and the maximal voluntary contraction pressure (mmHg) will be recorded using a validated intravaginal pressure device iEase XFT0010 (Sshenzhen XFT Medical Limited, Shenzhen, China). Vaginal manometric perineometry has demonstrated high intra-rater reliability for assessing PFM strength in women with PF disorders [53,54]. This variable was selected as the primary outcome for the a priori sample size calculation (Section 2.4) and directly addresses the primary mechanistic hypothesis of the trial (H1).

#### 2.7.2. Secondary Outcome Measures

- PFM function

PFM function will be evaluated using the PERFECT assessment scheme, developed and validated by Laycock and Jerwood [55]. The following components will be assessed according to standardized procedures: Power (maximal voluntary contraction graded 0–5 using the modified Oxford scale), Endurance (duration of a sustained maximal voluntary contraction, up to 10 s), Repetitions (number of repeated sustained contractions at the same intensity, up to 10), and Fast contractions (number of rapid 1 s contractions with full relaxation between efforts, up to 10). All digital palpation assessments will be performed by the same trained physiotherapist using a standardized assessment protocol, thereby eliminating inter-rater variability. The individual PERFECT components (Power, Endurance, Repetitions, and Fast contractions) will be recorded and analysed separately as secondary outcomes. No aggregate or total PERFECT score will be calculated.

Objective assessment of PFM activation will be performed using intracavitary electromyography (iEMG), a valid and reliable method for assessing PFM neuromuscular activation in women with PF dysfunction, including UI [56]. Maximum and mean voluntary contraction amplitudes will be recorded using the NeuroTrac^®^ MyoPlus2 Pro system (Verity Medical Ltd., Hampshire, UK) with a vaginal intracavitary electrode and a surface reference electrode. To improve between-session comparability and reduce the influence of electrode placement and reinsertion variability, iEMG amplitudes will be normalized within each assessment session. Specifically, EMG activity recorded during each contraction will be expressed as a percentage of the maximum voluntary contraction obtained during the same assessment session (%MVC). Absolute amplitudes in µV will be retained for descriptive purposes, whereas normalized %MVC values will be used for longitudinal comparisons across baseline, post-intervention, and follow-up assessments.

B. Urinary incontinence patterns and impact

UI-related symptoms and voiding patterns will be assessed using a 3-day voiding diary, a widely accepted and validated clinical tool that captures urinary frequency, leakage episodes, and symptom severity in daily life contexts [57].

UI severity and its impact on quality of life will be assessed using the International Consultation on Incontinence Questionnaire–Short Form (ICIQ-SF). The ICIQ-SF is a validated and widely used instrument in older adults. Total scores range from 0 to 21, with higher scores indicating greater symptom severity [35].

C. Abdominal muscle function

Abdominal muscle morphology and activation will be assessed using ultrasound imaging to evaluate inter-recti distance and transversus abdominis (TrA) activation. Measurements will be performed with a portable ultrasound device (General Electric Logiq E V2) equipped with a linear transducer, and values will be expressed in millimeters (mm). Participants will be positioned in a supine position with the LL flexed to minimize lumbar lordosis and abdominal wall tension. Inter-recti distance will be measured with the transducer placed transversely at standardized locations 1 cm above and 1 cm below the umbilicus. Measurements will be obtained under two conditions: at rest and during forced exhalation, corresponding to the activation phase of diaphragmatic breathing. The difference between conditions will be used to quantify changes in rectus abdominis separation.

Bilateral TrA activation will be assessed with the transducer positioned transversely along the mid-axillary line, midway between the iliac crest and the costal margin. Measurements will be performed at rest and during forced exhalation, and changes in muscle thickness between conditions will be used as an indicator of TrA activation. Ultrasound imaging has been shown to be a valid and reliable method for assessing abdominal muscle morphology and activation patterns in clinical and research settings [58,59].

D. Gait and aerobic capacity

Gait speed will be assessed using the 4-Meter Walk Test (4MWT) at usual and maximal walking speeds. To minimize acceleration effects, participants will start walking 0.5 m before the first marker and continue walking 0.5 m beyond the end marker. The time required to complete the central 4 m will be recorded, and gait speed will be calculated in meters per second (m/s) [60]. The 4MWT is a widely used and validated measure of functional mobility in older adults, demonstrating good reliability and strong clinical relevance [61].

Aerobic capacity will be evaluated with the Six-Minute Walking Test (6MWT) following standardized guidelines [62]. Gait biomechanics during walking will be assessed using Runscribe^TM^ inertial sensors (Scribe Labs, Moss Beach, CA, USA) placed on both feet, providing spatiotemporal parameters (speed, cadence, stride length, and gait cycle duration for the left and right foot) [63].

E. Lower-Limb muscle power

LL muscle power will be assessed using a Chronojump^®^ contact platform through vertical jumps tests, including the Countermovement Jump (CMJ) and the Abalakov Jump (ABK), which are sensitive to changes in muscular power in older adults [64,65].

LL muscle power will also be evaluated using a Chronojump^®^ linear encoder during two resistance exercises (squat and leg extension). Participants will perform one familiarization repetition followed by two sets of five repetitions for each exercise, with a one-minute rest between sets. All repetitions will be executed with maximal intentional concentric velocity. The Chronojump^®^ system has demonstrated high concurrent validity for displacement and velocity measurements in older adults (r > 0.94) [66]. All assessments will be conducted under standardized safety conditions, including close supervision by trained personnel and the use of protective measures when necessary. Participants presenting pain, dizziness, instability, or any contraindication to maximal effort testing will not perform the corresponding test.

F. Physical function of upper and lower limb

Functional LL performance will be assessed using the Five Times Sit-to-Stand (5tSTS) and the Thirty-Second Sit-to-Stand (30sSTS) tests, both validated and reliable measures of functional strength and physical performance in older adults [61,67,68]. Upper-limb muscle strength will be evaluated using 30 s Arm Curl Test, performed bilaterally, with the number of completed repetitions recorded for each arm [67].

G. Maximal isometric muscle strength

Maximal isometric muscle strength will be assessed using a Chronojump^®^ traction force sensor dynamometer (Chronojump^®^ Boscosystem, Barcelona, Spain), fixed to rigid structures to ensure mechanical stability. Force output will be recorded in newtons (N) and analyzed using the corresponding software (Chronojump^®^ Boscosystem, version 2.3.0-2629-x64).

The same standardized testing positions, dynamometer attachment points, and body alignment will be maintained across all assessments to ensure comparability within-participant over time. Force values are reported in newtons (N) and are not intended to represent joint torque. Isometric strength will be assessed bilaterally for the following muscle groups: knee extension, hip flexion, hip extension, hip abduction, hip adduction, and trunk (spine) extension. For each muscle group, participants will first perform one brief familiarization contraction (≈1 s) followed by two maximal isometric contractions of 5 s, with a 1 min rest between trials. Standardized testing positions and verbal instructions will be used across participants, and the order of muscle group testing will be counterbalanced to minimize fatigue effects. The mean value of the two maximal trials will be used for statistical analysis. For bilateral muscle groups, the left and right limbs be assessed, recorded, and analysed separately; therefore, no average bilateral value will be calculated. Detailed descriptions of the standardized testing positions, participant stabilization, dynamometer placement, testing sequence, and measurement procedures for each muscle group are provided in Supplementary Material S1. Participants will be stabilized using standardized body positioning and manual supervision when required to minimize compensatory trunk and pelvic movements during testing.

Upper-limb maximal isometric strength will be assessed using a digital handgrip dynamometer (Takei TKK 5401, Takei Scientific Instruments Co., Ltd., Tokyo, Japan). Participants will perform three maximal contractions with each hand, and the mean value will be used for analysis. Handgrip strength will be expressed in kilograms (kg) and has demonstrated high reliability in older adult populations [69].

H. Balance performance

Balance performance will be assessed using clinical and instrumental measures. Proactive balance will be evaluated with the Functional Reach Test [70,71], and dynamic balance with the Timed Up and Go test [72].

Static balance will be assessed using the Modified Romberg Test (participants will be held for 10 s without compensatory movements of the upper limbs or trunk) [61] and single-leg stance [73].

Additionally, static posturography will be used to analyze center-of-pressure (COP) displacement under different sensory conditions using the NedSVE/IBV^®^ system on a Dinascan/IBV force platform. The following COP variables will be collected: sway area (mm^2^) and maximum anteroposterior (AP) and mediolateral (ML) displacement (mm). Static posturography have demonstrated good clinical utility and moderate-to-high test–retest reliability in bipedal standing tasks under different sensory conditions [74,75].

I. Fear of falling

Fear of falling will be assessed using the short Falls Efficacy Scale–International (FES-I). In the present study, a shortened and validated Spanish version will be used, which assesses confidence in performing daily 7 activities associated with balance demands and fall risk. Higher total scores indicate greater concern about falling. This version has demonstrated adequate psychometric properties in Spanish-speaking populations [76]. Total scores will be used for statistical analysis.

J. Quality of life

Health-related quality of life will be assessed using the 36-Item Short Form Health Survey (SF-36), which evaluates eight domains of physical and mental health. Domain scores range from 0 to 100, with higher scores indicating better perceived health status and quality of life [77].

K. Psychological and social well-being

Self-esteem will be assessed using the Rosenberg Self-Esteem Scale (RSES), a widely used measure of global self-esteem. The scale consists of 10 items rated on a 4-point Likert scale, with total scores ranging from 10 to 40, and higher scores indicating higher self-esteem [78].

L. Physical Activity Questionnaire-Short Form (IPAQ-SF)

Physical activity levels will be assessed using the IPAQ-SF, a validated self-report questionnaire which records physical activity performed during the previous 7 days. Data will be processed according to the official IPAQ scoring protocol and expressed as total physical activity (MET-min/week). Participants will also be classified into the standard IPAQ physical activity categories (low, moderate, or high). Data cleaning and truncation procedures will follow the official IPAQ data processing guidelines. The IPAQ-SF will be administered at baseline (T0) and at the 6-month follow-up assessment (T2).

M. Body weight and composition

Body weight will be measured in kilograms (kg). Body composition parameters, including fat mass and lean mass percentages, will be assessed using bioelectrical impedance analysis (BIA) with a validated analyzer (Tanita^®^ BC-545N MA, Tokyo, Japan).

N. Height

Height will be measured to the nearest 0.1 cm using a calibrated stadiometer (SECA 217, Seca GmbH & Co., Hamburg, Germany), with participants standing barefoot in an upright position.

#### 2.7.3. Adherence, Safety and Co-Interventions

Before each assessment session, participants will be screened for any new medical conditions or contraindications that could compromise the safe performance of the assessments. Maximal jump tests (CMJ and ABK) will only be performed when participants are considered clinically fit by the supervising physiotherapist, as CMJ has been demonstrated to be a safe and valid assessment of LL muscle power in community-dwelling older adults [79,80].

Any assessment or exercise session will be immediately interrupted if the participant develops chest pain, severe dyspnea, dizziness, syncope, acute musculoskeletal pain, neurological symptoms, or any other clinical sign judged by the supervising physiotherapist to compromise safe participation. Participants may also stop any assessment or exercise session at their own request.

Adherence to the intervention will be systematically monitored throughout the study period using a structured session-by-session attendance register. A minimum attendance threshold of 80% of scheduled sessions will be required to classify a participant as adherent, consistent with criteria applied in comparable exercise-based trials in older adults. Reasons for any missed session will be documented by the supervising physiotherapist to distinguish between voluntary withdrawal, health-related absence, and logistical barriers, thereby enabling a more nuanced interpretation of adherence patterns across groups and time points.

AE will be recorded prospectively using a standardized monitoring log completed by the supervising physiotherapist at the end of each session. AE will be defined as any unintended sign, symptom, or condition occurring during or within 48 h of a session, regardless of causal attribution to the intervention. These will include musculoskeletal complaints (e.g., joint pain, muscle strain), cardiovascular symptoms (e.g., dizziness, chest discomfort, unusual dyspnea), PF-related symptoms (e.g., increased urinary leakage or pelvic pain following exercise), and any fall or near-fall event occurring within the study context. AE will be classified by severity (mild, moderate, severe) and by relationship to the intervention (unrelated, possibly related, probably related, definitely related). Serious adverse events (SAE) will be reported immediately to the principal investigator and to the Ethics Committee of the Catholic University of Valencia in accordance with applicable regulatory requirements. Participants experiencing an SAE will be temporarily or permanently withdrawn from the intervention at the discretion of the supervising clinician and will continue to be included in the intention-to-treat analysis wherever feasible. Participants who are temporarily withdrawn may resume participation once symptoms have resolved and, when clinically indicated, after reassessment by the supervising physiotherapist and medical clearance.

Co-interventions with the potential to influence study outcomes will be monitored throughout the intervention and follow-up periods using a structured self-reported log completed by participants at each assessment time point. At the post-intervention assessment (T1), participants will report any relevant co-interventions occurring during the intervention period (T0–T1), whereas at the follow-up assessment (T2), they will report co-interventions occurring during the follow-up period (T1–T2). Co-interventions of interest will include any physiotherapy treatment targeting the LL, spine, or PF; formal PFM rehabilitation programmes delivered outside the study context; and any initiation, discontinuation, or modification of pharmacological treatments known to affect urinary function, neuromuscular performance, or fall risk (e.g., anticholinergics, alpha-blockers, diuretics, benzodiazepines, or antihypertensives). The occurrence and nature of co-interventions will be reported descriptively and considered as potential confounding variables during data interpretation, particularly in cases where marked between-group imbalances are identified.

### 2.8. Statistical Analysis

#### 2.8.1. Data Analysis Plan

All statistical analyses will be performed using IBM SPSS Statistics (version 29) and R (version 4.3, R Core Team, 2024). All tests will be two-tailed, with statistical significance set at *p* ≤ 0.05. Descriptive statistics (means, standard deviations, frequencies, and percentages, as appropriate) will be reported for all sociodemographic, clinical, and outcome variables at each assessment time point.

Analyses will follow the intention-to-treat (ITT) principle, including all randomized participants in the groups to which they were originally allocated, regardless of intervention adherence or study completion. As a sensitivity analysis, a per-protocol analysis will also be conducted including only participants who attended ≥ 80% of scheduled sessions and completed all assessment time points.

The primary analysis will be conducted using likelihood-based estimation within the cLDA framework, which incorporates all available observations under the missing-at-random (MAR) assumption. Missing data patterns, including Little’s MCAR test, will be explored descriptively but will not determine the primary analytical approach. Multiple imputation by chained equations (MICE) will be performed as a pre-specified sensitivity analysis to evaluate the robustness of the primary findings.

The primary analysis will use linear mixed-effects models (LMM) treating all three assessment time points (T0, T1, T2) as repeated observations of the outcome variable, following a constrained longitudinal data analysis (cLDA) framework. In accordance with the cLDA approach, baseline means are constrained to be equal across randomized groups, reflecting the randomization assumption while modelling T0 as the first repeated outcome. The model will include the following fixed effects: group (four levels: Control, EX, EXVERB, EXVERBPSY), time (three levels: T0, T1, T2), and their interaction (group × time). Participant will be included as a random effect (random intercept). The primary inferential interest is placed on the group × time interaction, evaluated at T1 (post-intervention, Week 18) and T2 (follow-up, Week 44). The cLDA model is robust to missing data under the missing-at-random (MAR) assumption and does not require sphericity. As a pre-specified sensitivity analysis, an ANCOVA model including baseline values as covariates will also be performed for T1 and T2 to evaluate the robustness of the primary cLDA findings [81]. The within-participant covariance of repeated measurements will be modelled using an unstructured covariance matrix. Model assumptions will be verified through inspection of residual plots, Q-Q plots, and formal tests of normality (Shapiro–Wilk) and homoscedasticity (Levene) applied to model residuals. If distributional assumptions are substantially violated, non-parametric alternatives (Friedman test for within-group comparisons; Mann–Whitney U test for between-group comparisons at each time point) will be used as sensitivity analyses, with results from both approaches reported.

For the planned comparisons and multiplicity controls, when a significant group × time interaction is found for the primary endpoint (α = 0.05, unadjusted), the following five pre-specified pairwise contrasts will be conducted simultaneously within a single testing family, with sequential Holm–Bonferroni correction applied across all five contrasts at each time point [82]: EXVERBPSY vs. Control at T1; EXVERB vs. Control at T1; EX vs. Control at T1; EXVERBPSY vs. EXVERB at T1 and EXVERB vs. EX at T1. Contrasts 1–3 evaluate the superiority of each active intervention arm over the educational control, directly addressing H1. Contrasts 4–5 evaluate the incremental contribution of each sequentially added component—PSY support and PFM activation instructions, respectively—consistent with the additive design of the trial. The same family of five contrasts will be repeated at T2 to evaluate the persistence of effects at the 6-month follow-up, addressing H3. Between-group effect sizes will be reported as Cohen’s d with 95% confidence intervals for each planned pairwise contrast, and as partial eta-squared (η^2^p) with 95% confidence intervals for the overall group × time interaction F-test. The same family of planned pairwise comparisons will also be applied to secondary outcomes. For each outcome analysed, sequential Holm–Bonferroni correction will be applied across the five pre-specified pairwise contrasts at each time point to control the family-wise error rate within that outcome [82].

To formally evaluate the dose–response pattern hypothesized in H2, a pre-specified linear trend analysis will be conducted using orthogonal polynomial contrasts applied to the group factor within the LMM framework. The four groups will be assigned contrast weights reflecting the sequential addition of intervention components and their expected incremental dose: Control = −3, EX = −1, EXVERB = +1, EXVERBPSY = +3. These weights correspond to the standard orthogonal polynomial contrast coefficients for four equally spaced ordered groups [40]. This analysis therefore assumes equally spaced incremental treatment effects across the four study arms and is intended to test the prespecified hypothesis of a monotonic additive dose–response trend, rather than implying that the additional intervention components are biologically or clinically equivalent. A statistically significant positive linear contrast at T1 will be interpreted as confirmatory evidence supporting a monotonic dose–response relationship across the additive intervention arms, consistent with H2. Results at T2 will be interpreted as exploratory, addressing the persistence of the dose–response pattern at follow-up. The effect size for the linear trend will be reported as r^2^contrast = Fcontrast/(Fcontrast + dferror). This analysis will be conducted for the primary endpoint and, as exploratory analyses, for the pre-specified secondary outcomes.

All secondary and exploratory outcomes will be analysed using the same LMM framework. Sequential Holm–Bonferroni correction will be applied across the five pre-specified pairwise contrasts for each secondary outcome, consistent with the primary analysis. However, no global family-wise error rate correction will be applied across secondary outcomes. Results will be presented with adjusted *p*-values for the planes pairwise comparisons, Cohen’s d effect sizes with 95% confidence intervals, and η^2^p for global F-tests. Findings from the prespecified secondary outcomes will be interpreted as supportive rather than confirmatory, whereas exploratory outcomes will be interpreted as hypothesis-generating.

To address H4, pre-specified exploratory association analyses will be conducted within the intervention groups combined (EX, EXVERB, and EXVERBPSY; *n* ≈ 90). To reduce potential regression-to-the-mean bias, baseline-adjusted residualised change scores will be used instead of raw change scores. Residualised change scores will be obtained as the residuals from linear regression models in which each post-intervention (T1) or follow-up (T2) value is regressed on its corresponding baseline (T0) value. Separate residualised change scores will therefore be adjusted for the T1 (post-intervention) and T2 (follow-up) assessments, each adjusted for its corresponding baseline (T0) value. For the Short FES-I, lower scores indicate improvement; therefore, more negative residualised values will reflect greater reductions in fear of falling.

Three sequential analyses will be performed: (1) Pearson or Spearman correlations between residualised psychological change scores and intervention adherence (percentage of sessions attended); (2) correlations between residualised psychological change scores and residualised change scores in the primary endpoint (Δmaximal voluntary contraction pressure assessed by manometric perineometry) and prespecified secondary outcomes at T1 and T2; and (3) hierarchical multiple regression analyses conducted within the intervention groups, in which group allocation (EX, EXVERB, EXVERBPSY) is entered in Step 1 and residualised psychological change scores are added in Step 2, with residualised change in the primary endpoint and, separately, each secondary outcome entered as the dependent variable. The increment in R^2^ (ΔR^2^) will be reported as the indicator of additional variance explained beyond group assignment.

All H4 analyses are designated as exploratory and hypothesis-generating. No adjustment for multiple comparisons will be applied, and findings will be interpreted cautiously with emphasis on effect estimates, 95% confidence intervals, and the consistency of observed associations rather than on statistical significance alone. These analyses do not constitute a formal mediation analysis and do not permit causal inference about psychological mechanisms.

In addition, pre-specified exploratory subgroup analyses will examine whether intervention effects on PFM function and UI symptom severity (ICIQ-SF) differ according to UI subtype (stress, urgency, or mixed), classified prior to randomization as described in Section 2.3. Subgroup analyses will be conducted by adding a three-way interaction term (group × time × UI subtype) to the primary LMM. Given the substantially reduced statistical power within each subtype subgroup, results will be interpreted as hypothesis-generating rather than confirmatory, and no family-wise error rate correction will be applied.

Effect sizes will be reported using partial eta squared (η^2^p) for global F-tests, Cohen’s d for pairwise between-group comparisons at each time point, and Cohen’s d with 95% confidence intervals for pre-to-post change scores within groups. Effect sizes will be interpreted using conventional benchmarks (small: d = 0.20; medium: d = 0.50; large: d = 0.80).

In addition to statistical significance, the clinical relevance of intervention effects will be evaluated using established MCIDs where available. Specifically, the ICIQ-SF will be interpreted using its published MCID (2.52 points) [83]. Four outcomes without well-established MCIDs in community-dwelling older women, including the TUG [84] and the Short FES-I, clinical relevance will be interpreted using effect sizes, 95% confidence intervals, and comparison with previously published intervention studies [85].

#### 2.8.2. Data Management

All data will be stored in a password-protected electronic database and double-checked for accuracy and completeness by a second researcher. Personal identifying information will be stored separately from research data using participant ID codes, and all analyses will be conducted on anonymized datasets. Access to the final dataset will be restricted to principal investigator and authorized research team members. Data will be securely archived for a minimum of five years following study completion, in accordance with applicable institutional and national regulations. Given the low-risk nature of the intervention, no independent data monitoring committee (DMC) has been established.

## 3. Ethics and Dissemination

### 3.1. Auditing

Given the low-risk nature of the intervention and the academic scope of this trial, no independent auditing procedures are planned. The principal investigator will oversee study conduct, ensure adherence to the protocol, and perform periodic internal reviews to monitor data quality and ethical compliance.

### 3.2. Ethics Approval

This study protocol has received ethical approval from the Ethics Committee of the Catholic University of Valencia (reference: UCV/2022-2023/123). The study will be conducted in accordance with the principles of the Declaration of Helsinki and applicable national and international ethical standards.

All interventions will comply with the ethical guidelines of the Official College of Physiotherapists of the Valencian Community (ICOFCV). Prior to enrolment, participants will receive written information about the study objectives, procedures, potential benefits, and risks. Written informed consent will be obtained from all participants before participation.

Participants may withdraw from the study at any time without consequences. Any discontinuation due to AE or clinical deterioration will be documented, and participants will be included in the analysis according to the intention-to-treat principle.

Any important protocol modifications affecting the study design, eligibility criteria, interventions, outcomes, statistical analysis, or administrative aspects will be submitted for approval to the Ethics Committee of the Catholic University of Valencia before implementation and, where applicable, will also be updated in the ClinicalTrials.gov registry.

Personal data will be stored in encrypted files and processed exclusively for research purposes, in compliance with Organic Law 3/2018 on the Protection of Personal Data and Guarantee of Digital Rights.

### 3.3. Consent for Publication

Consent for publication is not applicable, as no identifiable personal data will be published. All results will be reported at the group level.

### 3.4. Dissemination Policy

Study findings will be disseminated through publication in peer-reviewed scientific journals and presentations at national and international conferences. A summary of the main results will be made available to participants upon request. Authorship will comply with ICMJE guidelines, and no professional writers will be involved.

### 3.5. Ancillary and Post-Trial Care

Given the low-risk nature of the intervention, no specific ancillary or post-trial care is planned. In the unlikely event of harm related to study participation, participants will be covered by the institutional insurance of the Catholic University of Valencia, in accordance with Spanish legislation.

### 3.6. Access to Data

Access to the final trial dataset will be restricted to the principal investigator and authorized members of the research team. All data will be anonymized prior to analysis. There are no contractual restrictions on data access.

## 4. Discussion

This study protocol describes a randomized controlled trial designed to evaluate the effectiveness of a multidisciplinary intervention combining PMT, PFM activation, and PSY support in community-dwelling older women with UI and increased fall risk. The intervention aims to improve balance, fear of falling, functional capacity, PFM control, and UI-related symptoms, with greater benefits expected in the most comprehensive intervention arm.

Previous evidence supports the effectiveness of MTP for fall prevention and functional improvement in older adults, as well as the role of exercise-based interventions in the management of UI [86,87,88]. Balance training and progressive strength or power-oriented exercises have been consistently associated with improvements in postural control and mobility. In parallel, PSY approaches addressing fear of falling, emotional regulation, and treatment adherence may enhance the effectiveness and sustainability of physical interventions [89].

In this context, PMT can be considered a specific and optimized form of MTP, incorporating high-velocity resistance exercises to enhance functional performance and fall-related outcomes in older adults. While exercise-based and PFM interventions have demonstrated benefits when applied independently, evidence regarding their combined and integrated application, particularly when supplemented with PSY strategies, remains limited [26,41,90].

This study seeks to address this gap by evaluating a comprehensive, feasible, and scalable intervention model aligned with the objectives of the Decade of Healthy Ageing (2021–2030). By addressing two highly prevalent and interrelated geriatric syndromes, this trial may contribute relevant evidence to inform multidisciplinary prevention strategies in both clinical and community settings.

In addition to its expected clinical benefits, the FIRE-WOMEN intervention may have important implications for healthcare services and public health strategies. By targeting two highly prevalent and interrelated geriatric syndromes, UI and fear of falling, this multidisciplinary approach may contribute to reducing healthcare utilization, preventing disability, delaying dependency, and supporting healthy ageing in the community. If effective, the intervention could represent a feasible model for integrating exercise-based rehabilitation and PSY support within community and primary healthcare settings.

### Limitations

The present protocol has several limitations that should be acknowledged.

(i) The active intervention groups receive substantially more participant–therapist contact than the educational control group (36 supervised exercise sessions vs. four monthly educational sessions), which may contribute to observed between-group differences beyond the specific intervention components. The control condition was intentionally designed to reflect realistic community practice rather than an attention-matched control, and the educational sessions provided to this group, covering healthy ageing, fall prevention, and pelvic floor health, may have improved participants’ knowledge and health-related behaviours, potentially affecting some outcomes, although they were not expected to produce the specific physiological adaptations associated with supervised exercise.
(ii) The sample size was calculated exclusively based on the primary endpoint (maximal voluntary contraction pressure assessed by manometric perineometry). Accordingly, this trial may not be adequately powered to detect smaller between-group differences in secondary outcomes or in the pre-specified subgroup analyses according to UI subtype. These analyses should therefore be considered exploratory and hypothesis-generating.
(iii) The study is restricted to community-dwelling older women, limiting generalizability to institutionalised populations or men.
(iv) Intracavitary EMG amplitudes will be normalised to within-session MVC (%MVC) to reduce inter-session variability; however, this approach does not fully eliminate variability arising from training-induced changes in MVC neuromuscular characteristics, and longitudinal iEMG findings should be interpreted with appropriate caution.
(v) Movement velocity during power training sessions will not be directly monitored; although the maximum intended velocity instruction and RPE-based intensity targets provide operational control of the training stimulus, confirmation that participants trained within the intended velocity range on a repetition-by-repetition basis is not possible.
(vi) Falls and near-falls will not be prospectively recorded during the intervention or follow-up period; future trials in this population should be adequately powered to include fall incidence as a primary endpoint alongside functional and psychological outcomes.
(vii) The a priori sample size was estimated using a repeated-measures ANOVA approximation in G*Power 3.1 [43], as dedicated LMM-specific power calculators are not readily available; under the assumed compound-symmetry covariance structure this approximation is considered conservative and reproducible, but represents a minor methodological limitation of the a priori calculation.

## 5. Conclusions

This study describes the FIRE-WOMEN trial, a four-arm additive randomized controlled trial designed to evaluate whether progressively adding PFM activation instructions and PSY support to a PMT programme provides additional benefits for PFM function, UI severity, balance performance, fear of falling, and physical function in community-dwelling older women.

The trial addresses two highly prevalent and interrelated geriatric syndromes through a multidisciplinary intervention that integrates physical, behavioural, and PSY components within a single rehabilitation programme. By incorporating a rigorous methodology, blinded outcome assessment, long-term follow-up, and a comprehensive evaluation of PF, functional, and psychosocial outcomes, this protocol aims to generate high-quality evidence on the effectiveness of this multidisciplinary intervention and its additive components.

The findings of the FIRE-WOMEN trial may contribute to the development of more comprehensive, evidence-based rehabilitation strategies for older women with UI and may support the implementation of multidimensional interventions aimed at improving PFM function, UI severity, balance performance, fear of falling, and quality of life.

## Figures and Tables

**Table 1:** Inclusion and exclusion criteria.

Inclusion Criteria	Exclusion Criteria
Women aged 60 to 80 years. Community-dwelling (non-institutionalized). Present UI after clinical diagnosis. Ability to walk independently, with or without assistive devices. Functional independence, defined as a Barthel Index score >90. Preserved cognitive function (MMSE score ≥25; GDS score ≥2 only if MMSE cannot be administered). Willingness to participate throughout the intervention and follow-up periods, with written informed consent provided. Not currently engaged in structured physical exercise programs within the previous 3 months Commitment to attend at least 80% of the scheduled intervention sessions.	Previous diagnosis of: (a) neurological disorders (e.g., Parkinson’s disease, stroke, Alzheimer’s disease); (b) severe musculoskeletal conditions limiting physical activity; (c) unstable cardiovascular disease (e.g., unstable angina, NYHA class III-IV heart failure), that could interfere with intervention or assessments. Surgery involving the PF, spine, hip, or knee points within the previous 6 months. Presence of pelvic organ prolapse grade II–IV. Current or recent (<3 months) participation in other structured exercise or intervention programs. Psychiatric or behavioural disorders that may interfere with adherence to the intervention. Ongoing use of medications known to significantly impair neuromuscular or cognitive function (e.g., strong muscle relaxants or sedatives). Inability to understand or follow basic instructions. Visual and/or hearing impairments that may hinder participation in the intervention or assessment.

Abbreviations: GDS: Global Deterioration Scale; MMSE: Mini-Mental State Examination.

**Table 2:** Multicomponent Exercise Program Schedule.

Program	Weeks 1–2	Weeks 3–7	Weeks 8–12	Weeks 13–18
Warm-up/ Mobility	Dynamic mobility (e.g., spine, shoulders, wrists, hips, knees) and general stretching	Similar to week 1–2	Similar to week 1–2	Similar to week 1–2
Balance	Bipedal stance on stable surface: feet together, semi-tandem and tandem (bilateral). Bipedal stance on unstable surface: feet together. Eyes open.	Bipedal stance on stable surface: feet together, semi-tandem and tandem (bilateral). Bipedal stance on unstable surface: feet together and semi-tandem (bilateral). Eyes open/closed.	Bipedal stance on stable surface: semi-tandem, tandem and unipedal (bilateral). Bipedal stance on unstable surface: semi-tandem and tandem (bilateral). Eyes open/closed.	Bipedal stance on stable surface: tandem and unipedal (bilateral). Bipedal stance on unstable surface: tandem and unipedal (bilateral). Eyes open/closed.
Power	Knee extension (seated) and hip extension (bipedal stance). 2 sets × 10 reps (60% intensity/iRPE: 2–3, fRPE: 5–6)	Exercises from Week 1–2. 2 sets × 8 reps (65% intensity)./iRPE: 3–4, fRPE: 5–6)	Exercises from Week 1–2. 3 sets × 8 reps (70% intensity)/iRPE: 3–4, fRPE: 5–6)	Exercises from Week 1–2. 3 sets × 8 reps (70% intensity)./iRPE: 4–5, fRPE: 5–6)
Strength	- Lateral trunk inclinations. - Squat with shoulder raises. - Standing hip abduction. - Standing shoulder abduction. 2 sets × 10 reps (60% intensity/RPE 5–6)	- Lateral trunk inclinations. - Squat with shoulder raises. - Standing hip abduction. - Standing shoulder abduction. 2 sets × 10 reps (65% intensity/RPE 6–7).	- Squat with shoulder raises. - Standing hip abduction. - Standing shoulder abduction. 3 sets × 10 reps (70% intensity/RPE 7–8).	- Squat with shoulder raises. - Standing hip abduction. - Standing shoulder abduction. 3 sets × 8 reps (80% intensity/RPE 8–9).
Aerobics (endurance)	Varied gait patterns with speed/direction changes (e.g., military march, lateral/backward walking, lunges, jumps) (65% intensity/RPE 11)	Exercises from Week 1–2. (65–75% intensity/RPE 11–13)	Exercises from Week 1–2. (70–80% intensity/RPE 13–14)	Exercises from Week 1–2. (75–85% intensity/RPE 14–15)
Cool-down/Relaxation	Diaphragmatic breathing. Global stretching.	Exercises from Week 1–2.	Exercises from Week 1–2.	Exercises from Week 1–2.

Abbreviations: RPE: rating of perceived exertion; iRPE: initial rating of perceived exertion corresponding to the perceived exertion of the first repetition of the set; fRPE: final rating of perceived exertion corresponding to the perceived exertion at the end of the set.

**Table 3:** Balance exercise progression.

Weeks	Type of Surface	Balance Exercise Program and Dose
Weeks 1–2	Stable surface	- Bipedal stance with feet together and eyes open. 3 × 30 s (total 1.5 min). Last set with hands crossing the shoulders. - Bipedal stance with feet together in semi-tandem position (bilateral) and eyes open. 3 × 30 s each side (total 3 min). Last set with hands crossing the shoulders. - Bipedal stance with feet together in tandem position (bilateral) and eyes open. 3 × 30 s each side (total 3 min). Last set with hands crossing the shoulders.
	Unstable surface	Bipedal stance with feet together and eyes open 3 × 30 s each side (total 3 min). Last set with hands crossing the shoulders.
Weeks 3–7	Stable surface	- Bipedal stance with feet together in semi-tandem position (bilateral) and eyes open. 3 × 30 s each side (total 3 min). Last set with hands crossing the shoulders. - Bipedal stance with feet together in tandem position (bilateral) and eyes open. 3 × 30 s each side (total 3 min). Last set with hands crossing the shoulders. - Bipedal stance with feet together and eyes closed. 2 × 30 s each side (total 1 min)
	Unstable surface	- Bipedal stance with feet together in semi-tandem position (bilateral) and eyes open. 3 × 30 s each side (total 3 min). Last set with hands crossing the shoulders. - Bipedal stance with feet together and eyes closed. 2 × 30 s each side (total 1 min). Last set with hands crossing the shoulders.
Weeks 8–12	Stable surface	- Bipedal stance with feet together in tandem position (bilateral) and eyes open. 2 × 30 s each side (total 2 min). Last set with hands crossing the shoulders. - Unipedal posture with eyes open. 2 × 30 s each side (total 2 min) - Bipedal stance with feet together in semi-tandem position (bilateral) and eyes closed. 2 × 30 s each side (total 2 min)
	Unstable surface	- Bipedal stance with feet together in semi-tandem position (bilateral) and eyes open. 2 × 30 s each side (total 2 min) Last set with hands crossing the shoulders. - Bipedal stance with feet together in tandem position (bilateral) and eyes open. 2 × 30 s each side (total 2 min). Last set with hands crossing the shoulders. - Bipedal stance with feet together in semi-tandem position (bilateral) and eyes closed. 2 × 30 s each side (total 2 min)
Weeks 13–18	Stable surface	- Bipedal stance with feet together in tandem position (bilateral) and eyes open. 2 × 30 s each side (total 2 min). Both sets with hands crossing the shoulders. - Bipedal stance with feet together in tandem position (bilateral) and eyes closed. 2 × 30 s each side (total 2 min) - Unipedal posture with eyes open. 2 × 30 s each side (total 2 min). Last set with hands crossing the shoulders. - Unipedal posture with eyes closed. 2 × 30 s each side (1 min)
	Unstable surface	- Bipedal stance with feet together in tandem position (bilateral) and eyes open. 2 × 30 s each side (total 2 min). Both sets with hands crossing the shoulders. - Bipedal stance with feet together in tandem position (bilateral) and eyes closed. 2 × 30 s each side (total 2 min) - Unipedal posture with eyes open. 2 × 30 s each side (total 2 min). Last set with hands crossing the shoulders.

Abbreviations: min: minutes; s: seconds.

**Table 4:** Power training progression.

Weeks	Exercises	Volume	Intensity
Weeks 1–2	Seated knee extension Standing hip extension	2 sets × 10 reps	iRPE: 2–3, fRPE: 5–6
Weeks 3–7	Seated knee extension Standing hip extension	2 sets × 8 reps	iRPE: 3–4, fRPE: 5–6
Weeks 8–12	Seated knee extension Standing hip extension	3 sets × 8 reps	iRPE: 3–4, fRPE: 5–6
Weeks 13–18	Seated knee extension Standing hip extension	3 sets × 8 reps	iRPE: 4–5, fRPE: 5–6

Abbreviations: iRPE: initial rating of perceived exertion corresponding to the perceived exertion of the first repetition of the set; fRPE: final rating of perceived exertion corresponding to the perceived exertion at the end of the set.

**Table 5:** Description of power exercises.

Exercise	Setup	Execution	Key Cues	Common Errors/Safety
Knee extension	Sit upright on a chair, elastic band looped around the working ankle and anchored under the chair leg; knee flexed ~90°.	Fast concentric knee extension to near full extension, 1 s isometric hold, controlled eccentric return (~2 s).	“Sit tall”; “extended fast, return slow”; “Knee tracks straight.”	Avoid hyperextension; reduce resistance if trunk moves; stop if knee pain > 2/10.
Standing hip extension	Stand facing a stable support (wall, bar, or chair) with feet hip-width apart. Elastic band looped around the working ankle and anchored to the opposite ankle or a low fixed point, with light pre-tension. Slight hip hinge with neutral pelvis, ribs down, core engaged; working knee slightly flexed.	Fast concentric hip extension moving the leg backward ~20–30° without lumbar sway, followed by a 1 s isometric hold. Controlled eccentric return to the starting position over ~2 s.	“Push the floor back with your heel.” “Move from the hip, not the low back.” “Keep the chest quiet and the core engaged.”	Lumbar extension or arching (reduce range, maintain rib control). Toe external rotation (maintain neutral alignment). Loss of balance or leg swinging (reduce band tension or use support). Avoid end-range lumbar extension; discontinue if sharp or radiating posterior hip pain occurs.

Abbreviation: s: seconds.

**Table 6:** Prescription parameters in strength exercises.

Weeks	Exercises	Volume	Intensity
Weeks 1–2	Lateral trunk inclinations Squat with shoulder press Standing hip abduction Standing shoulder abduction (scapular plane, 30°)	2 sets × 10 reps	fRPE 5–6
Weeks 3–7	Lateral trunk inclinations Squat with shoulder press Standing hip abduction Standing shoulder abduction (scapular plane, 30°)	2 sets × 10 reps	fRPE 6–7
Weeks 8–12	Squat with shoulder press Standing hip abduction Standing shoulder abduction (scapular plane, 30°)	3 sets × 10 reps	fRPE 7–8
Weeks 13–18	Squat with shoulder press Standing hip abduction Standing shoulder abduction (scapular plane, 30°)	3 sets × 8 reps	fRPE 8–9

Abbreviations: fRPE: final rating of perceived exertion corresponding to the perceived exertion at the end of the set.

**Table 7:** Description of strength exercises.

Exercise	Setup	Execution	Key Cues	Common Errors/Safety
Lateral trunk inclinations (side bends)	Stand tall with feet hip-width apart and knees slightly flexed. Hold the elastic band handle in the working hand; the opposite hand rests lightly on the pelvis. Core gently braced with ribs stacked over pelvic plane.	From an upright position, perform a controlled eccentric lateral trunk flexion by sliding the hand down the thigh, keeping shoulders level and avoiding rotation. Return concentrically to neutral using the contralateral trunk musculature.	“Grow tall, then tip—don’t twist.” “Keep the pelvis quiet; move through the ribs.”	Hip shift, lumbar extension, or trunk rotation. Perform only within a pain-free range and avoid end-range compression if spinal symptoms occur.
Squat with shoulder press	Stand with feet shoulder-width apart and toes slightly externally rotated. Anchor the elastic band under the feet and hold the handles at shoulder height with elbows slightly forward and trunk braced.	Perform a controlled eccentric squat to a comfortable depth. During the concentric phase, extend the hips and knees and simultaneously press the arms overhead to a soft lockout. Lower the arms back to shoulder level over ~2 s before the next repetition.	“Knees track, heels down; stand then reach tall.” “Keep ribs down during the press.”	Knee valgus, heel lift, lumbar extension during the press, or initiating the press before full LL extension. Maintain a pain-free shoulder range and discontinue if lumbar extension is required.
Standing hip abduction (straight-leg)	Stand facing a stable support. Elastic band looped around both ankles. Feet parallel, pelvis neutral, knees slightly flexed, trunk upright.	Move the working leg laterally ~20–30° without pelvic elevation or trunk lean. Hold for 1 s at end range, then return to the starting position over ~2 s under control. Repeat and switch sides.	“Engage the core; slide the leg sideways.” “Keep toes and kneecap facing forward.”	Trunk lean, pelvic hike, toe external rotation, or snapping at the hip. Avoid lateral trunk compensation and stop if sharp lateral hip pain occurs.
Standing shoulder abduction in the scapular plane	Stand upright with neutral spine. Hold elastic band handles with arms positioned ~30° anterior to the frontal plane (scapular plane), thumbs slightly up.	Raise both arms in the scapular plane to approximately 90° of shoulder elevation without shrugging. Hold for 1 s, then lower the arms over ~2 s under control.	“Ribs down, neck long.” “Lift in the scapular plane—don’t shrug.”	Shoulder shrugging, rib flare, internal rotation of the arms, or lifting beyond symptom-free range. Maintain scapular plane positioning and stop if impingement pain occurs.

**Table 8:** Description of aerobic exercises.

Phase	Main Aerobic Tasks	Duration	Intensity	Progression Criteria
Weeks 1–2	Forward/backward walking, side stepping, marching tasks	10 min.	65% HRmax. Borg RPE 11	Familiarization with movement patterns
Weeks 3–7	Multidirectional gait, tempo changes, coordinates arm-leg tasks	10 min.	65–75% HRmax. Borg RPE 11–13	Increased movement complexity and tempo
Weeks 8–12	Accelerations/decelerations, obstacle negotiation, dance-based sequences	10 min.	70–80% HRmax. Borg RPE 13–14	Reduced external support and increased coordination demands
Weeks 13–18	Dual-task gait, reactive stepping, complex gait sequences	10 min.	75–85% HRmax. Borg RPE 14–15	Increased cognitive and motor complexity

Abbreviations: min: minutes; HRmax: maximum heart rate; RPE: rating of perceived exertion.

**Table 9:** Description of the PSY intervention sessions.

Session	Core Focus	Key Contents/Techniques	Home Practice
1	Understanding emotions	Components of emotional experience (thoughts, behaviours, and physiological sensations) and their interaction.	Emotional experience logs
2	Emotional awareness	Analysis of learned emotional responses will be discussed through four key principles: (1) individuals learn from past experiences, approaching what feels pleasant and avoiding what feels unpleasant; (2) behavioural patterns often develop to prevent exposure to potential discomfort; (3) some behaviours initially used to manage intense emotions become habitual over time (e.g., avoiding eye contact); and (4) although emotion-driven behaviours may appear adaptive, repeated reliance on them can reinforce maladaptive cycles that become insensitive to context.	Practice of experimental exercises
3	Understanding thoughts	Identification of emotion-related thoughts; cognitive distortions; cognitive reappraisal and cognitive defusion techniques.	Continued cognitive practice
4	Understanding behaviors	Adaptive vs. maladaptive behaviours; emotional avoidance and safety behaviours; planning of alternative responses.	Practice and alternative behaviors
5	Perspective-taking and acceptance	Self-observation from an integrated perspective; acceptance and compassion-focused exercises.	Perspective-taking practice
6	Self-acceptance and self-compassion	Four A’s model: (1) Attention, (2) Appreciation, (3) Affection, and (4) Acceptance; compassionate-self exercise; program summary	Continue self-compassion practice

## Data Availability

No datasets were generated or analyzed during the current study protocol. Data generated during the future randomized controlled trial will be available from the corresponding author upon reasonable request and in accordance with ethical and privacy regulations.

## Supplementary Materials

The following supporting information can be downloaded at: https://www.mdpi.com/article/10.3390/healthcare14162467/s1, Table S1: SPIRIT 2025 schedule of enrolment, interventions, and assessments; Table S2: SPIRIT 2025 checklist; Supplementary Material S1: Standardized testing positions and procedures for maximal isometric strength assessment.

## Abbreviations

The following abbreviations are used in this manuscript:

ABK	Abalakov
ACSM	American College of Sports Medicine
ACT	Acceptance and Commitment Therapy
AE	Adverse Event
AP	Anteroposterior
BIA	Bioelectrical Impedance Analysis
CMJ	Countermovement Jump
CONSORT	Consolidated Standards of Reporting Trials
DMC	Data monitoring Committee
EX	Exercise
EXVERB	Exercise plus Verbal Pelvic Floor Muscle Activation Instructions
EXVERBPSY	Exercise plus Verbal Pelvic Floor Muscle Activation Instructions and Psychoeducation
FES-I	Falls Efficacy Scale International
GDS	Global Deterioration Scale
GLMM	Generalized Linear Mixed Model
HRmax	Maximum Heart Rate
ICIQ-SF	International Consultation on Incontinence Questionnaire-Short Form
iEMG	Intracavitary Electromyography
ITT	Intention-to-Treat
LL	Lower Limb
LMM	Linear Mixed Model
MAR	Missing At Random
MCID	Minimal Clinically Important Difference
MICE	Multiple Imputation by Chained Equations
MMSE	Mini-Mental State Examination
ML	Mediolateral
MTP	Multicomponent Training Program
NYHA	New York Heart Association
PF	Pelvic Floor
PFM	Pelvic Floor Muscle
PMT	Power-based Multicomponent Training
PSY	Psychoeducation/psychoeducational Intervention
RPE	Rating of Perceived Exertion
RSES	Rosenberg Self-Esteem Scale
SAE	Serious Adverse Event
SF-36	36-Item Short Form Health Survey
SPIRIT	Standard Protocol Items: Recommendations for Interventional Trials
TrA	Transversus Abdominis
UI	Urinary Incontinence
4MWT	Four-Meter Walk Test
6MWT	Six-Minute Walk Test
5tSTS	Five Times Sit To Stand
30sSTS	Thirty Seconds Sit To Stand
