# Supplementary material for: Power-Based Multicomponent Training, Pelvic Floor Muscle Activation Instructions, and Psychoeducation for Urinary Incontinence, Balance and Fear of Falling in Older Women (FIRE-WOMEN): Study Protocol for a Randomized Controlled Trial
Source: Healthcare (Basel). 2026 Aug 10;14(16):2467. doi: 10.3390/healthcare14162467 (PMC13512584; doi:10.3390/healthcare14162467)
Supplement: Supplementary file 1 [file healthcare-14-02467-s001.zip › Supplementary Material S1.pdf]

Power-Based Multicomponent Training, Pelvic Floor Muscle Activation Instructions, and Psychoeducation for Urinary Incontinence, Balance and Fear of Falling in Older Women (FIRE-WOMEN): Study Protocol for a Randomized Controlled Trial

Maximal isometric strength: **testing positions and procedures**

Knee extension: Participants will be seated with the hip and knee flexed at approximately 90°, with the trunk supported against the backrest. An ankle strap with a posterior attachment will be placed on the distal leg and connected via a chain to the force sensor, which will be anchored to a fixed point posterior to the limb, aligned horizontally. From a starting position of 90° knee flexion, participants will be instructed to perform a maximal isometric knee extension, following the verbal cue *"extend the knee and lift the foot without flexing the hip."*

Trunk extension: Participants will be seated with 90° hip flexion, without trunk support against the backrest. A harness will be adjusted to each participant's torso circumference and placed around the trunk.The force sensor will be attached at chest level and connected via chains to a fixed anchor point positioned in front of the participant. Participants will be instructed to perform a maximal isometric trunk extension, following the verbal cue *"extend your trunk backwards without pushing with your feet."*

Hip flexion: Participants will be seated with the hip and knee flexed at approximately 90°, with the trunk supported against the backrest. A strap will be placed around the distal thigh and connected via a chain to the force sensor anchored to a fixed point posterior and distal to the tested limb. Participants will be instructed to perform a maximal isometric hip flexion, following the verbal cue *"lift the knee upwards without using your hands."*

Hip extension: Participants will stand upright, stabilizing themselves by lightly holding a wall or stall bars with the hand ipsilateral to the tested limb. An ankle strap with an external attachment will be connected via a chain to the force sensor anchored to a fixed point on the wall. The starting position will include approximately 10° of hip flexion. Participants will

be instructed to perform a maximal isometric hip extension, following the verbal cue *“keep the knee extended and push the leg backwards.”*

Hip abduction: Participants will stand upright, stabilizing themselves with the contralateral hand. An ankle strap with an internal attachment will be connected to the force sensor anchored to a fixed point on the wall anterior to the participant. Starting from approximately 10° of hip adduction, participants will be instructed to perform a maximal isometric hip abduction, following the verbal cue *“keep the knee extended and move the leg outward.”*

Hip adduction: Participants will stand upright, stabilizing themselves with the hand ipsilateral to the tested limb. An ankle strap with an external attachment will be connected to the force sensor anchored to a fixed point on the wall. Starting from approximately 10° of hip abduction, participants will be instructed to perform a maximal isometric hip adduction, following the verbal cue *“keep the knee extended and bring the leg toward the midline.”*

The same standardized protocol, participant positioning, stabilization procedures, dynamometer placement, testing sequence, and verbal instructions will be used for all participants and all assessment sessions.
