# Supplementary material for: Power-Based Multicomponent Training, Pelvic Floor Muscle Activation Instructions, and Psychoeducation for Urinary Incontinence, Balance and Fear of Falling in Older Women (FIRE-WOMEN): Study Protocol for a Randomized Controlled Trial
Source: Healthcare (Basel). 2026 Aug 10;14(16):2467. doi: 10.3390/healthcare14162467 (PMC13512584; doi:10.3390/healthcare14162467)
Supplement: Supplementary file 1 [file healthcare-14-02467-s001.zip › Table S1. SPIRIT TABLE (2o).pdf]

Table S1. SPIRIT 2025 Schedule of enrolment, interventions, and assessments.

**Domain**	**Instrument / Measure**	**Screening**	**Baseline (T0)**	**Week 18 Post-intervention (T1)**	**Week 44 Follow-up (T2)**
**ENROLMENT**					
Eligibility screening	Clinical diagnosis of UI, MMSE (≥25) (or GDS (≤2) if MMSE cannot e administered), Barthel Index (>90)	X			
Cognitive function	MMSE ≥25 (routine assessment). GDS ≥2 only if MMSE cannot be administered. If both assessments are performed, the MMSE result determines eligibility.	X			
Informed consent	Written consent form	X			
Randomization	Computer-generated sequence (Sealed Envelope™)		X		
**SOCIODEMOGRAPHIC & CLINICAL DATA**					
Age, sex, educational level	Structured interview		X		
Obstetric and gynaecological history	Structured interview		X		
Comorbidities and medication use	Health history interview		X		
Physical activity level	IPAQ-SF		X		
Anthropometrics (weight, height, BMI)	Stadiometer (SECA 217), scales		X	X	X
Body composition (fat mass, lean mass)	BIA (Tanita® BC-545N MA)		X	X	X
**INTERVENTIONS**					
EX group (PMT only)	Power based Multicomponent exercise programme			Weeks 1–18	

EXVERB group (PMT + PFM cues)	Exercise + verbal PFM activation instructions			Weeks 1–18	
EXVERBPSY group (PMT + PFM + PSY)	Exercise + PFM activation + 6 PSY sessions			Weeks 1–18	
Control group	4 informational sessions (geriatric syndrome prevention)			Weeks 1–18	
**PRIMARY OUTCOMES**					
Pelvic floor strength					
PFM maximal contraction pressure (mmHg)	Manometric perineometry (iEase XFT0010)		X	X	X
**SECONDARY OUTCOMES**					
Pelvic floor function					
PFM strength (modified Oxford scale, 0–5)	PERFECT scheme (Laycock & Jerwood)		X	X	X
PFM neuromuscular activation (μV)	Intracavitary EMG — NeuroTrac MyoPlus2 Pro		X	X	X
Urinary symptoms and voiding patterns	3-day bladder diary		X	X	X
UI severity and impact	ICIQ-SF (0–21)		X	X	X
Abdominal muscle function					
Inter-recti distance (mm)	Ultrasound imaging (GE Logiq E V2)		X	X	X
Transversus abdominis activation (mm thickness)	Ultrasound imaging (GE Logiq E V2)		X	X	X
Gait and aerobic capacity					
Gait speed (m/s) – usual and maximal	4- Meter Walk Test (4MWT)		X	X	X
Aerobic capacity (m)	Six-Minute Walk Test (6MWT)		X	X	X
Spatiotemporal gait parameters	Runscribe™ inertial sensors		X	X	X
Lower-limb muscle power					

Vertical jump power — CMJ and ABK (cm)	Chronojump® contact platform		X	X	X
LL power during squat and leg extension (W, m/s)	Chronojump® linear encoder		X	X	X
Physical function of upper and lower limbs			X	X	X
LL power in a funcitonal task	Five Times Sit-to-Stand (5tSTS) (s)		X	X	X
LL endurance strength	Thirty-Second Sit-to-Stand (30sSTS) (reps)		X	X	X
Upper-limb endurance strength	30-Second Arm Curl Test (reps, bilateral)		X	X	X
Maximal isometric muscle strength					
Isometric strength — knee ext., hip flex./ext./abd./add., trunk ext. (N)	Chronojump® traction force sensor dynamometer		X	X	X
Handgrip strength (kg)	Digital dynamometer (Takei TKK 5401)		X	X	X
Balance			X	X	X
Proactive balance	Functional Reach Test (cm)		X	X	X
Dynamic balance	Timed Up and Go Test (s)		X	X	X
Static balance	Modified Romberg Test (10 s)		X	X	X
Static balance	Single-leg stance test (s)		X	X	X
Static posturography — sway area (mm^2), AP/ML displacement (mm)	NedSVE/IBV® — Dinascan/IBV force platform		X	X	X
Fear of falling					
Falls-related self-efficacy	Falls Efficacy Scale–International (FES-I), short Spanish version		X	X	X
Health-related quality of life	SF-36 (8 domains, 0–100)		X	X	X
Self-esteem	Rosenberg Self-Esteem Scale (RSES, 10–40)		X	X	X

**SAFETY AND ADHERENCE**					
Adverse events	Structured monitoring log (session-by-session)			Weeks 1-18 (continuous monitoring)	
Session attendance	Attendance register (target ≥80%)			Weeks 1-18 (continuous monitoring)	
Co-interventions	Self-reported log (PT, PFM rehab, UI medication changes)			Weeks 1-18 (interval-based monitoring; covers T0-T1)	Weeks 18-44 (interval-based monitoring; covers T1-T2)
Physical activity levels during follow-up	IPAQ-SF		X		X

Note. ICIQ-SF: International Consultation on Incontinence Questionnaire–Short Form; GDS: Global Deterioration Scale; MMSE: Mini-Mental State Examination; BIA: Bioelectrical Impedance Analysis; PFM: Pelvic Floor Muscle; iEMG: intracavitary electromyography; PERFECT: Power, Endurance, Repetitions, Fast contractions, Every Contraction Timed; CMJ: Countermovement Jump; ABK: Abalakov Jump; 4MWT: Four-Meter Walk Test; 6MWT: Six-Minute Walk Test; 5tSTS: Five Times Sit-to-Stand; 30sSTS: Thirty-Second Sit-to-Stand; FES-I: Falls Efficacy Scale–International; SF-36: Short Form-36 Health Survey; RSES: Rosenberg Self-Esteem Scale; AP: anteroposterior; ML: mediolateral; LL: lower limb; T0: baseline; T1: post-intervention (week 18); T2: follow-up (week 42); EX: exercise only; EXVERB: exercise + verbal PFM cues; EXVERBPSY: exercise + verbal PFM cues + psychoeducation; PSY: psychoeducational sessions.

REFERENCE:

Chan AW, Boutron I, Hopewell S, Moher D, Schulz KF, Collins GS, et al. SPIRIT 2025 statement: updated guideline for protocols of randomised trials. BMJ. 2025;389:e081477. doi:10.1136/bmj-2024-081477.
