# Supplementary material for: Power-Based Multicomponent Training, Pelvic Floor Muscle Activation Instructions, and Psychoeducation for Urinary Incontinence, Balance and Fear of Falling in Older Women (FIRE-WOMEN): Study Protocol for a Randomized Controlled Trial
Source: Healthcare (Basel). 2026 Aug 10;14(16):2467. doi: 10.3390/healthcare14162467 (PMC13512584; doi:10.3390/healthcare14162467)
Supplement: Supplementary file 1 [file healthcare-14-02467-s001.zip › Table S2. SPIRIT 2025- CHECKLIST.pdf]

Table S2: SPIRIT 2025 expanded checklist detailing items to address in randomized trial protocols, based on Explanation and Elaboration document

Cite as: Chan A-WC, Boutron I, Hopewell S et al. SPIRIT 2025 statement: updated guideline for protocols of randomised trials. BMJ 2025;389:e081477. doi:10.1136/bmj-2024-081477

SPIRIT 2025			Addressed on page number / section
**Section / Topic**	**No**	**Description**	
**Administrative information**			
Title and structured summary	1a	Title stating the trial design, population, and interventions, with identification as a protocol	Title page 1 (Power-based multicomponent training, pelvic floor muscle activation instructions, and psychoeducation for urinary incontinence, balance and fear of falling in older women (FIRE-WOMEN): Study protocol for a randomized controlled trial)
	1b	Structured summary of trial design and methods, including items from the World Health Organization Trial Registration Data Set	Abstract (Background/Objectives, Methods, Expected results, Conclusions; ClinicalTrials.gov NCT06839040).
Protocol version	2	Version date and identifier	Title page 1 (Protocol version: Version 2.0, July 2026)
Roles and responsibilities	3a	Names, affiliations, and roles of protocol contributors	Title page 1
	3b	Name and contact information for the trial sponsor	Title page 1
	3c	Role of trial sponsor and funders in design, conduct, analysis, and reporting of trial; including any authority over these activities	Title page 1
	3d	Composition, roles, and responsibilities of the coordinating site, steering committee, endpoint adjudication committee, data management team, and other individuals or groups	Title page 1

		overseeing the trial, if applicable	
**Open science**			
Trial registration	4	Name of trial registry, identifying number (with URL), and date of registration. If not yet registered, name of intended registry	Page 1
Protocol and statistical analysis plan	5	Where the trial protocol and statistical analysis plan can be accessed	Study protocol (Section 2.8.1, Data analysis plan)
Data sharing	6	Where and how the individual de-identified participant data (including data dictionary), statistical code, and any other materials will be accessible	Data Availability Statement (page 33)
Funding and conflicts of interest	7a	Sources of funding and other support (e.g., supply of drugs)	Funding statement (page 33)
	7b	Financial and other conflicts of interest for principal investigators and steering committee members	Conflicts of Interest statement (page 33)
Dissemination policy	8	Plans to communicate trial results to participants, healthcare professionals, the public, and other relevant groups (e.g., reporting in trial registry, plain language summary, publication)	Section 3.4. Dissemination policy (Ethics and dissemination) (page 31)
**Introduction**			
Background and rationale	9a	Scientific background and rationale, including summary of relevant studies (published and unpublished) examining benefits and harms for each intervention	Introduction

	9b	Explanation for choice of comparator	Introduction; Section 2.6 (Control group intervention)
Objectives	10	Specific objectives related to benefits and harms	Introduction (page 4)
**Methods: Patient and public involvement, trial design**			
Patient and public involvement	11	Details of, or plans for, patient or public involvement in the design, conduct, and reporting of the trial	Not applicable.
Trial design	12	Description of trial design including type of trial (e.g., parallel group, crossover), allocation ratio, and framework (e.g., superiority, equivalence, non-inferiority, exploratory)	Section 2.1 Study design (page 5)
**Methods: Participants, interventions, and outcomes**			
Trial setting	13	Settings (e.g., community, hospital) and locations (e.g., countries, sites) where the trial will be conducted	Section 2.2. Study population (page 5)
Eligibility criteria	14a	Eligibility criteria for participants	Section 2.2.1. Selection criteria (page 5)
	14b	If applicable, eligibility criteria for sites and for individuals who will deliver the interventions (e.g., surgeons, physiotherapists)	Section 2.2. Study population (page 5); Section 2.6.2. General aspects of the exercise protocol (page 10)
Intervention and comparator	15a	Intervention and comparator with sufficient details to allow replication including how, when, and by whom they will be administered. If relevant, where additional materials describing the intervention and comparator (e.g., intervention manual) can be accessed	Sections 2.6. interventions (page 9); Figures 2–7; Tables 2–8; Supplementary Material S1

	15b	Criteria for discontinuing or modifying allocated intervention/comparator for a trial participant (e.g., drug dose change in response to harms, participant request, or improving/worsening disease)	Section 2.7.3. Adherence, safety and co-interventions (page 26)
	15c	Strategies to improve adherence to intervention/comparator protocols, if applicable, and any procedures for monitoring adherence (e.g., drug tablet return, sessions attended)	Sections 2.6.2 (page 10) and 2.7.3 (page 26)
	15d	Concomitant care that is permitted or prohibited during the trial	Section 2.7.3. Adherence, safety and co-interventions (page 26)
Outcomes	16	Primary and secondary outcomes, including the specific measurement variable (e.g., systolic blood pressure), analysis metric (e.g., change from baseline, final value, time to event), method of aggregation (e.g., median, proportion), and time point for each outcome	Section 2.7. Outcome measures (page 23); Table S1
Harms	17	How harms are defined and will be assessed (e.g., systematically, non-systematically)	Section 2.7.3. Adherence, safety and co-interventions (page 26); Table S1
Participant timeline	18	Time schedule of enrolment, interventions (including any run-ins and washouts), assessments, and visits for participants. A schematic	Figure 7 (page 22); Table S1

		diagram is highly recommended (see Figure).	
Sample size	19	How sample size was determined, including all assumptions supporting the sample size calculation	Section 2.4. Sample size calculation (page 7)
Recruitment	20	Strategies for achieving adequate participant enrolment to reach target sample size	Section 2.3. Recruitment process and screening (page 7)
**Methods: Assignment of interventions**			
Randomization:			
Sequence generation	21a	Who will generate the random allocation sequence and the method used	Section 2.5. Randomization and blinding (page 8)
	21b	Type of randomization (simple or restricted) and details of any factors for stratification. To reduce predictability of a random sequence, other details of any planned restriction (e.g., blocking) should be provided in a separate document that is unavailable to those who enroll participants or assign interventions	Section 2.5. Randomization and blinding (page 8)
Allocation concealment mechanism	22	Mechanism used to implement the random allocation sequence (e.g., central computer/telephone; sequentially numbered, opaque, sealed containers), describing any steps to conceal the sequence until interventions are assigned	Section 2.5. Randomization and blinding (page 8)
Implementation	23	Whether the personnel who will enroll and those who will assign participants to the	Section 2.5. Randomization and blinding (page 8)

		interventions will have access to the random allocation sequence	
Blinding	24a	Who will be blinded after assignment to interventions (e.g., participants, care providers, outcome assessors, data analysts)	Section 2.5. Randomization and blinding (page 8)
	24b	If blinded, how blinding will be achieved and description of the similarity of interventions	Section 2.5. Randomization and blinding (page 8)
	24c	If blinded, circumstances under which unblinding is permissible, and procedure for revealing a participant's allocated intervention during the trial	Not applicable
**Methods: Data collection, management, and analysis**			
Data collection methods	25a	Plans for assessment and collection of trial data, including any related processes to promote data quality (e.g., duplicate measurements, training of assessors) and a description of trial instruments (e.g., questionnaires, laboratory tests) along with their reliability and validity, if known. Reference to where data collection forms can be accessed, if not in the protocol	Section 2.7. Outcome measures (page 23); Supplementary Material S1
	25b	Plans to promote participant retention and complete follow-up, including list of any outcome data to be collected for participants who discontinue or deviate from intervention protocols	Section 2.7.3. Adherence, safety and co-interventions (page 26)

Data management	26	Plans for data entry, coding, security, and storage, including any related processes to promote data quality (e.g., double data entry; range checks for data values). Reference to where details of data management procedures can be accessed, if not in the protocol	Section 2.8.2. Data management (page 30)
Statistical methods	27a	Statistical methods used to compare groups for primary and secondary outcomes, including harms	Section 2.8.1. Data analysis plan (page 27)
	27b	Definition of who will be included in each analysis (e.g., all randomized participants), and in which group	Section 2.8.1. Data analysis plan (ITT and per-protocol analyses) (page 27)
	27c	How missing data will be handled in the analysis	Section 2.8.1. Data analysis plan (page 27)
	27d	Methods for any additional analyses (e.g., subgroup and sensitivity analyses)	Section 2.8.1. Data analysis plan (page 27)
**Methods: Monitoring**			
Data monitoring committee	28a	Composition of data monitoring committee (DMC); summary of its role and reporting structure; statement of whether it is independent from the sponsor and funder; conflicts of interest and reference to where further details about its charter can be found, if not in the protocol. Alternatively, an explanation of why a DMC is not needed	Section 3.1. Auditing (page 30)
	28b	Explanation of any interim analyses and stopping	Section 2.7.3. Adherence, safety and co-interventions (page 26)

		guidelines, including who will have access to these interim results and make the final decision to terminate the trial	
Trial monitoring	29	Frequency and procedures for monitoring trial conduct. If there is no monitoring, give explanation	Section 3.1. Auditing (page 30)
**Ethics**			
Research ethics approval	30	Plans for seeking research ethics committee/institutional review board approval	Section 3.2. Ethics approval (page 30)
Protocol amendments	31	Plans for communicating important protocol modifications to relevant parties	Section 3.2. Ethics approval (page 30)
Consent or assent	32a	Who will obtain informed consent or assent from potential trial participants or authorized proxies, and how	Section 3.2. Ethics approval (page 30)
	32b	Additional consent provisions for collection and use of participant data and biological specimens in ancillary studies, if applicable	Section 3.3. Consent for publication (page 31)
Confidentiality	33	How personal information about potential and enrolled participants will be collected, shared, and maintained in order to protect confidentiality before, during, and after the trial	Section 3.2. Ethics approval (page 30); Section 3.6. Access to data (page 31)
Ancillary and post-trial care	34	Provisions, if any, for ancillary and post-trial care, and for compensation to those who suffer harm from trial participation	Section 3.5. Ancillary and post-trial care (page 31)
